# Mutant PRPF8 Causes Widespread Splicing Changes in Spliceosome Components in Retinitis Pigmentosa Patient iPSC-Derived RPE Cells

**DOI:** 10.3389/fnins.2021.636969

**Published:** 2021-04-29

**Authors:** Ángeles Arzalluz-Luque, Jose Luis Cabrera, Heli Skottman, Alberto Benguria, Arantxa Bolinches-Amorós, Nicolás Cuenca, Vincenzo Lupo, Ana Dopazo, Sonia Tarazona, Bárbara Delás, Miguel Carballo, Beatriz Pascual, Imma Hernan, Slaven Erceg, Dunja Lukovic

**Affiliations:** ^1^Department of Applied Statistics, Operations Research and Quality, Universitat Politècnica de València, València, Spain; ^2^Genomics Unit, Centro Nacional de Investigaciones Cardiovasculares (CNIC). Madrid, Spain; ^3^Faculty of Medicine and Health Technology, Tampere University, Tampere, Finland; ^4^Stem Cells Therapies in Neurodegenerative Diseases Lab, Research Center Principe Felipe, Valencia, Spain; ^5^National Stem Cell Bank-Valencia Node, Research Center Principe Felipe, Valencia, Spain; ^6^Department of Physiology, Genetics and Microbiology, University of Alicante, Alicante, Spain; ^7^Unit of Genetics and Genomics of Neuromuscular and Neurodegenerative Disorders, Centro de Investigación Príncipe Felipe (CIPF), Valencia, Spain; ^8^Rare Diseases Joint Units, IIS La Fe-CIPF, Valencia, Spain; ^9^Unitat de Genética Molecular, Hospital de Terrassa, Terrassa, Spain; ^10^Department of Neuroregeneration, Institute of Experimental Medicine, Czech Academy of Sciences, Prague, Czechia; ^11^Retinal Degeneration Lab, Research Centre Principe Felipe, Valencia, Spain

**Keywords:** iPSC, RPE, RNA-Seq, retinitis pigmentosa, pre-mRNA splicing, alternative splicing, PRPF8

## Abstract

Retinitis pigmentosa (RP) is a rare, progressive disease that affects photoreceptors and retinal pigment epithelial (RPE) cells with blindness as a final outcome. Despite high medical and social impact, there is currently no therapeutic options to slow down the progression of or cure the disease. The development of effective therapies was largely hindered by high genetic heterogeneity, inaccessible disease tissue, and unfaithful model organisms. The fact that components of ubiquitously expressed splicing factors lead to the retina-specific disease is an additional intriguing question. Herein, we sought to correlate the retinal cell-type-specific disease phenotype with the splicing profile shown by a patient with autosomal recessive RP, caused by a mutation in pre-mRNA splicing factor 8 (PRPF8). In order to get insight into the role of PRPF8 in homeostasis and disease, we capitalize on the ability to generate patient-specific RPE cells and reveal differentially expressed genes unique to RPE cells. We found that spliceosomal complex and ribosomal functions are crucial in determining cell-type specificity through differential expression and alternative splicing (AS) and that PRPF8 mutation causes global changes in splice site selection and exon inclusion that particularly affect genes involved in these cellular functions. This finding corroborates the hypothesis that retinal tissue identity is conferred by a specific splicing program and identifies retinal AS events as a framework toward the design of novel therapeutic opportunities.

## Introduction

Retinitis pigmentosa (RP), the most common form of hereditary retinal dystrophies, is a progressive blinding disease that currently lacks effective therapies (Hartong et al., 2006). Typically, light-sensing photoreceptors and the underlying retinal pigment epithelium (RPE), responsible for photoreceptor homeostasis, are the primary degenerated cells. RP is highly genetically heterogeneous with over 80 disease-causing genes identified to date^1^. Mutations in splicing proteins are described to account altogether as a second most common cause of autosomal dominant RP (adRP) (Sullivan et al., 2006).

Splicing reaction is a process of excision of non-coding intervening sequences, introns, and exon ligations resulting in coding capacity diversification of a limited number of expressed genes with > 90% of 20,000 human protein-coding genes producing multiple mRNA isoforms. This stepwise reaction is performed by spliceosome, a multisubunit ribonucleoprotein complex with the core components uridine-rich small non-coding nuclear RNAs (snRNAs, U1, U2, U4, U5, and U6) and associated proteins that assemble on pre-mRNA to catalyze the excision of introns (Wahl et al., 2009). Mutations in seven genes coding for pre-mRNA processing factors known as PRPFs (PRPF8, PRPF31, PRPF3, PRPF4, and PRPF6), SNRNP200, and PAP-1 have been described to cause adRP (Sullivan et al., 2006).

While these genes are expressed ubiquitously in all tissues and are highly conserved among eukaryotes, it remains unclear why mutations thereof exhibit exclusively retinal phenotype. It has been proposed that *PRPF* mutations might cause global splicing dysregulation and affects only retina because of retinal enhanced splicing activity (Tanackovic et al., 2011). Alternatively, a retina-specific splicing code could confer tissue-specific transcripts that are altered by mutations in splicing factors and therefore cause the disease.

PRPF8 is a 220-kDa, evolutionary highly conserved protein described to play a central role in spliceosomal catalytic activity (Grainger and Beggs, 2005; Galej et al., 2013). Mutations in the C-terminal tail have been described to cause adRP (RP13, OMIM600059) (McKie et al., 2001) while mutations in the N-terminal part have recently been found in glaucoma patients. Mutations in PRPF8, unlike PRPF31, show in the majority of cases complete penetrance, with marked disease progression, exhibiting moderate phenotype with residual vision up to their fifth decade or severe clinical phenotypes initiating in the first decade of life (Martinez-Gimeno et al., 2003; Maubaret et al., 2011; Escher et al., 2018). The degree of severity is proposed to be related to the type of mutation (Escher et al., 2018). Mouse models, *Prpf8*-H2309P with knocked-in missense mutation, showed only mild phenotype in RPE ultrastructure only at 2 years of age, without any loss of ERG signal (Graziotto et al., 2011). Therefore, these animal models are insufficient and human models that recapitulate the clinical phenotype are required to decipher the mechanism of disease.

Native human retina is a largely inaccessible tissue that cannot provide enough research material. Moreover, it is non-expandable due to its postmitotic state. The generation of induced pluripotent stem cells (iPSCs) from accessible tissue like patient’s skin, and coaxing them toward retinal tissue, offers an unprecedented opportunity to study the disease and also find new therapies. Moreover, the advantage of the iPSC-based models is that they recapitulate the disease-causing mutation and patient’s genetic complexity, essential for complex and late-onset diseases. The iPSCs can be directed toward both photoreceptors and RPE cells, capturing their morphology and function (Hirami et al., 2009; Reichman et al., 2014; Zhong et al., 2014) and have been used successfully in disease modeling (Lukovic et al., 2015, 2020; Shimada et al., 2017; Buskin et al., 2018). The identification of primary affected cell type in RP is hampered due to strong interplay between photoreceptor and RPE cells; namely, mutations of genes expressed in RPE lead to secondary photoreceptor degeneration; conversely, mutations in genes expressed in photoreceptors induce collateral RPE disruptions. This task is additionally obscured in PRPF-caused RP owing to their ubiquitous nature. Recent studies support RPE as likely primary affected cell type by PRPF mutations. Mouse models *Prpf3*-T49M, *Prpf8*-H2309P and *Prpf31*^+ ⁣/−^ showed moderate ultrastructural alterations including loss of basal infoldings, accumulation of amorphous deposits beneath the RPE and presence of vacuoles while neural retina remained intact (Graziotto et al., 2011). These morphological alterations observed in old mice are preceded by functional disruption in phagocytosis of photoreceptor outer segments (POS) and altered adhesion between RPE microvilli and photoreceptors 10 days upon birth (Farkas et al., 2014). Finally, in PRPF31-RP patient-derived photoreceptors and RPE, both cell types were altered, however; iPSC-RPE were most severely affected with ultrastructural and functional abnormalities. Furthermore, this study showed that spliceosome global dysregulation underlies disrupted RPE polarity, trans-epithelial resistance, phagocytosis, primary cilia length, and incidence in human and mouse genetic background (Buskin et al., 2018). The aforementioned studies prompted us to study PRPF8 pathomechanism in a cell-autonomous manner in patient-derived RPE cells.

Herein, we have generated RPE cells from reprogrammed dermal skin fibroblasts derived from a patient diagnosed with adRP and bearing a pathogenic mutation in *PRPF8* (NM_006445:c.6974_6994del) (Lukovic et al., 2017) in a heterozygous state (from here on: *Patient-RPE*) and patient’s non-affected tissue, i.e., fibroblasts (from here on: *Patient-fibroblasts*). An unrelated, non-affected individual was used as control (from here on: *Control-RPE* and *Control-fibroblasts*). We generated RNA sequencing (RNA-Seq) data for three independent experimental replicates per sample and performed a whole-transcriptome analysis where healthy control samples were compared to mutation-bearing cells, in order to depict retinal-specific transcriptional signature caused by the mutation.

## Materials and Methods

### Human Subjects

Research procedures followed the principles of the Declaration of Helsinki. The study was approved by the Research Ethical Committee of the Hospital of Terrassa and Valencian Ethics Committee CAEC (Comité Etico Autonomico de Estudios Clínicos), Valencia, Spain. Both subjects provided written informed consent after the nature of the study procedures had been fully explained.

### adRP Patient’s Clinical Phenotype

The patient reported a history of nyctalopia starting from the first decade of life. At the age of 20 the best-corrected visual acuity (BCVA) was 20/40 in the right eye and 20/30 in the left eye. Anterior segment examination showed minimal posterior subcapsular cataract in the right eye, being normal in the left eye. Fundus examination showed the typical RP changes, consisting in optic disk pallor, retinal vessel attenuation, and extensive bone spicule-like pigmentation extending from the vascular arcades toward the periphery. No abnormalities at the macula were detected. Humphrey visual field examination showed a severe visual field loss, with 10° central preserved and a peripheral remnant in the left eye. Electroretinography was abolished at that time.

At the age of 47, lens surgery had been performed in the right eye, so that BCVA was 20/40. In the right eye, she presented a moderate subcapsular cataract, being the BCVA 20/70. Fundus examination showed the typical RP changes. Spectral domain optical coherence tomography showed a complete absence of outer retinal bands corresponding to photoreceptors. Macular edema or epiretinal membrane was not detected in any moment of evolution.

### Cell Line Derivation and Differentiation

#### Fibroblast Cell Culture

Cryopreserved fibroblast cell lines derived from skin biopsies from the female RP patient (Patient-fibroblasts) and a healthy (BCVA40/40) man (Control-fibroblasts) were thawed and cultured in DMEM (10% FBS, 2 mM GlutaMAX, 50 U/ml penicillin and 50 mg/ml streptomycin) at 37°C under 5% CO_2_. Fibroblasts at passages 6–8 were used in this study. Samples were collected in triplicates, from independent cell cultures. Authentication of the fibroblast cell lines by microsatellite STR markers was performed to confirm the identity to the iPSC lines.

#### iPSC Cell Culture and Differentiation Toward RPE

Two iPSC lines were employed in this study (Supplementary Table 4). The iPSC line derived from the patient, clinically diagnosed with RP and molecularly genotyped previously, by reprogramming dermal fibroblasts using a non-integrative Sendai virus (Lukovic et al., 2017) has been employed. As control cell line, iPSC Ctrl1-FiPS4F1 (Spanish National Stem Cell Bank) obtained from a healthy individual by the same reprogramming strategy (Sendai virus, Cytotune, Thermo Fisher) was used. Both cell lines were induced to differentiate toward RPE following the previously published protocol (Lukovic et al., 2015; Hongisto et al., 2017). Briefly, the iPSCs were cultured in hESC medium (KO DMEM, KSR 20%, GlutaMAX 2 mM, non-essential amino acids 0.1 mM, β-mercaptoethanol 0.23 mM, basic FGF 10 ng/ml, and penicillin/streptomycin) on irradiated (45 Gy) human feeder cells (ATCC CRL2429) at 37°C under 5% CO_2_. When the colonies reached confluency, they were lifted and cultured in suspension in the absence of bFGF until the dark patches appeared. The dark patches were excised, trypsinized, and plated on collagen type IV/LN-521 coated plastic cell culture dishes where they acquired the RPE morphology within 4–8 weeks. RPE-like cells were passed through a 70-μm cell strainer in order to reach a homogeneous, highly pigmented RPE-like monolayer with characteristic uniform polygonal cell morphology. The differentiation experiments were performed in triplicates for both genotypes (*Patient-RPE* and *Control-RPE*). The samples were collected from each differentiation experiment (*n* = 3) and RNA extraction was performed.

#### Immunocytochemistry

Cells were fixed with 4% paraformaldehyde (PFA), washed twice in PBS, and placed in blocking solution (3% normal goat or donkey serum and 0.5% Triton-X 100 in PBS) for 1 h at room temperature. Cells were then incubated overnight at 4°C with one of the following primary antibodies: anti-PRPF8 (1: 200, Santa Cruz sc55534, Abcam ab79237), rabbit anti-CRALBP (1:250, Abcam), rabbit anti-ZO1 (1:50, Invitrogen), and mouse anti-Na/K-ATPase (1:100, Santa Cruz). The following day, cells were washed three to five times in PBS and incubated with an appropriate secondary antibody (1:500, Invitrogen). After secondary antibody incubation, nuclei were stained with 4′,6-diamidino-2-phenylindole, dihydrochloride (DAPI) (Life Technologies, #D1306) and washed three times in PBS. The coverslips were mounted using Vectashield Mounting Medium (Vector Lab, Burlingame, CA, United States) and imaged on Leica confocal microscope SP8.

### POS Phagocytosis Assay

Bovine rod POS were obtained from InVision BioResources (WA, United States) and labeled with FITC. Phagocytosis assay was performed as described previously (Lukovic et al., 2015). Briefly, iPSC-RPE cells were incubated with POS for 2 h/37°C, washed with PBS, and fixed by 4% PFA. After permeabilization by 0.1% Triton-X, phalloidin staining was performed and the samples were mounted with Vectashield mounting media. The samples were visualized by Leica confocal microscope SP8 using HCX PL APO lambda blue 63X/1.4 OIL objective. The quantification of POS particles was performed by imaging random fields from three different experiments in each condition. Five images were taken from each experiment. The relative intensity of internal POS labeling was quantified using Image J, and the pairwise comparison was performed using Student’s *t* test. Results are expressed as the mean ± SD. Values of *p* < 0.05 were considered statistically significant.

### Western Blot

Fibroblast cells were lysed in RIPA buffer (R0278 Sigma) containing a protease inhibitor cocktail (Roche), and total protein was quantified using a Bradford Reagent protein assay (B6916 Sigma-Aldrich). Protein lysates were denatured by 1X SDS Sample Buffer. The resulting samples were incubated at 95°C for 5 min. Protein samples (100 μg) were then separated on TGX Stain-FreeTM Gels (Bio-Rad) and electroblotted onto a PVDF membrane (Trans-Blot^®^ TurboTM Transfer Pack/Bio-Rad). Membranes were incubated in blocking buffer (5% non-fat dried milk diluted in TBS + 0.1% Tween) for 1 h at room temperature, washed three times in TBS + 0.1% Tween for 5 min, and incubated with primary antibody (PRPF8 Abcam ab79237) at 1:500 dilution in blocking buffer overnight at 4°C. Thereafter, blots were washed three times in TBS + 0.1% Tween and incubated with secondary HRP-conjugated antibody in blocking buffer for 45 min at RT. Blots were washed another five times and protein bands were visualized using SuperSignal West Pico PLUS (Thermo Scientific) on X-ray films. β-ACTIN (monoclonal, 1:4,000,000, Sigma-Aldrich A3854) was used as a loading control. HiMark Prestained Protein Standard (LC5699, Thermo) was used as a molecular weight standard.

### Transmission Electron Microscopy

RPE cells were fixed in 4% PFA and 2% glutaraldehyde in 0.1 M sodium phosphate buffer (pH 7.2–7.4) for 2 h, washed with the same buffer, and then postfixed in 1% OsO4 in PB. After gradual dehydration in ethanol series, the pieces were embedded in EPON 812. Semithin and ultrathin sections were obtained in an ultramicrotome (Leica Ultracut R, Leica Microsystem). After staining with lead citrate and uranil acetate, ultrathin sections were examined in a JEM-1400 Plus electron microscope (JEOL GmbH, München, Germany).

### RNA-Seq Library Preparation and Sequencing

High-quality total RNA (RIN > 7) from three independent experimental replicates of Patient-RPE, Control-RPE, Patient-fibroblasts, and Control-fibroblasts was extracted by RNeasy mini kit (Qiagen, Germany) and quantified by Agilent 2100 Bioanalyser. Total RNA (200 ng) was used to generate barcoded RNA-Seq libraries using the NEBNext Ultra RNA Library preparation kit (New England Biolabs). First, poly A + RNA was purified using poly-T oligo-attached magnetic beads followed by fragmentation and first and second cDNA strand synthesis. Next, cDNA ends were repaired and adenylated. The NEBNext adaptor was then ligated, followed by uracil excision from the adaptor and PCR amplification. The size of the libraries was checked using the Agilent 2100 Bioanalyzer and the concentration was determined using the Qubit^®^ fluorometer (Life Technologies). Libraries were sequenced on a HiSeq2500 (Illumina) to generate 60-base (sequencing batch 1) or 50-base (sequencing batch 2) single-end reads, to a mean depth of 100M reads/sample for one replicate for each condition (sequencing batch 1) and a mean depth of 20M reads/sample for the other two replicates (sequencing batch 2). FastQ files for each sample were obtained using CASAVA v1.8 software (Illumina).

### RNA-Seq Analysis

#### Read Mapping, Quantification and QC

Quality control assessments for raw RNA-Seq reads were performed using fastqc (version 11.5)^2^. TruSeq Illumina adapters (3′ adapters, sequence: AGATCGGAAG AGCACACGTCTGAACTCAGTCA) were removed using cutadapat (version 2.5, option –discard-trimmed activated, detected 2–5% reads with adapters) (Anders et al., 2015). Then, the STAR aligner (version 2.7.3a, median 89.3% reads mapped) was used for mapping the reads to the human genome (GRCh38.p7) using the ENSEMBL transcriptome (release 87) (Yates et al., 2020). Gene-level counts were obtained using HTSeq (Anders et al., 2015) (htseq-count, version 0.11.0, median 79.5% of reads assigned to genes). Mapping QC was performed using Qualimap (version 2.1.2) and MultiQC (version 1.7) (Ewels et al., 2016).

Gene-level counts were normalized using Trimmed Mean of M Values (TMM) (Robinson and Oshlack, 2010). RNA-Seq QC was performed using NOISeq (version 2.30.0) (Tarazona et al., 2015), removing features with mean expression < 1 Count per Million (CPM) across all samples. Batch effects between sequencing batches were then corrected using the ARSyN method (implemented in NOISeq) (Nueda et al., 2012). Principal Component Analysis (PCA) was performed using the prcomp() function included in the stats R package (version 3.6.3).

### Differential Expression and Functional Enrichment Analysis

Differential expression (DE) analysis was performed using NOISeqBio (Tarazona et al., 2015). Four contrasts were performed to obtain DE genes (*p* value <0.05—equivalent to NOISeq probability > 0.95—and log2FC ≥ 1) between all meaningful pairs of clinical conditions and cell types: (A) two within-cell type contrasts to compare clinical conditions for the same cell type, i.e., Control-Fibroblasts vs. Patient-Fibroblasts and Control-RPE vs. Patient-RPE; and (B) two within-condition contrasts to detect differences between cell types for the same clinical condition, i.e., Control-Fibroblasts vs. Control-RPE and Patient-Fibroblasts vs. Patient-RPE.

To better interpret the DE results, we used the information in the control and fibroblast contrasts to filter the results of the patient and RPE contrasts, respectively. In particular, in the within-cell type contrasts, DE genes found in the Control-Fibroblasts vs. Patient-Fibroblasts that were common to the Control-RPE vs. Patient-RPE were removed from the latter list (Supplementary Table 1). Since fibroblasts present no disease phenotype, any differences in expression between fibroblasts from both clinical conditions is expected to arise from technical effects and non-phenotype-related biological effects and is considered to be a source of interference for correct interpretation. In case of the within-condition contrasts, we considered only unique DE genes detected in the control (Control-fibroblasts vs. Control-RPE) and patient (Patient-fibroblasts vs. Patient-RPE) contrasts and removed common DE genes from both. In this manner, we address differences in expression related to encoding of the healthy RPE phenotype that are lost in the disease (DE in control contrast, and not DE in patient contrast) as well as expression changes that arise due to the RP disease and were not present when comparing healthy cells (DE in patient contrast, and not DE in control contrast).

Functional Enrichment analysis of these sets of DE genes was performed for Gene Ontology (GO) annotations [retrieved using the biomaRt (Durinck et al., 2005) R package version 2.42.1, GO database accessed December 2019 (Ashburner et al., 2000; Carbon et al., 2019)] using Fisher’s Exact Test and Benjamini–Hochberg’s multiple-testing correction method (significance threshold: adj *p* value <0.05).

### Alternative Splicing Event Analysis and Functional Enrichment of Alternatively Spliced Genes

Alternative splicing (AS) event analysis was performed for the same four contrasts as in DE analysis using SUPPA2 (version 2.3) (Lareau and Brenner, 2015). The RefSeq human transcriptome (RefSeq release 96) (O’Leary et al., 2016) was used to define splicing events via the generateEvents function in SUPPA2. We then mapped sequencing reads to the human genome (GRCh38.p13) using STAR [ref STAR] (version 2.7.3a) and the same RefSeq transcriptome and obtained isoform-level expression estimates using RSEM (version 1.3.0) (Yates et al., 2020). Transcripts per Million (TPM) values were then used to obtain a Percentage Spliced In (PSI) value per splicing event using the psiPerEvent function (SUPPA2). Next, differential splicing analysis was performed using the diffSplice function (SUPPA2) to obtain the PSI difference (dPSI) and *p* value for the splicing change in the four contrasts of interest.

In order to obtain alternatively spliced genes for each contrast, we performed several filtering steps. For the within-cell type contrasts, events with a significant splicing change (*p* value <0.05) in both the fibroblast (Control-Fibroblasts vs. Patient-Fibroblasts) and RPE (Control-RPE vs. Patient-RPE) contrasts were removed from the latter to preserve only splicing differences related to the disease retinal phenotype (Supplementary Table 2). In the case of the within-condition contrasts, we removed common alternatively spliced events from the significant events obtained in each of the two contrasts, i.e., Control-fibroblasts vs. Control-RPE and Patient-fibroblast vs. Patient-RPE. Hence, significant events unique to the control were considered to be associated to splicing differences encoding the healthy phenotype that are then lost in the patient, while unique patient events were interpreted as arising as a result of the disease and the PRPF8 mutation. In this, case, however, we also considered common significant events changing the sign of dPSI between contrasts.

Functional enrichment analysis of differentially spliced genes was performed as described in the previous section. As our list of splicing-related genes, we performed a text-mining search of GO terms containing the words “mRNA”, “pre-mRNA”, “splicing”, “spliceosome”, “spliceosomal”, and “rRNA” (total GOs: 106) and selected all genes annotated for at least one of these GOs (total genes: 987). A complete list of all considered GO terms is provided in Supplementary File 9. We note that some false-positive AS-related GOs may arise from this type of search; however, it is designed to be as comprehensive as possible in order to provide interesting candidates for the RP disease. Annotations are subsequently manually checked, and only truly AS-related genes are discussed and considered in the manuscript.

## Results

### Patient-Specific iPSC-RPE Cells Reveal Normal Cellular Phenotype

We previously identified an adRP patient with the heterozygous mutation in *PRPF8* (NM_006445:c.6974-6994del, p.Val2325_Glu2331del) (Martinez-Gimeno et al., 2003) (Supplementary Figure 1). This mutation affects the C-terminal region of the PRPF8 within a region required for interaction with EFTUD2 and SNRNP200 (Pena et al., 2007). This 21-base pair deletion results in Val2325 to Glu2331 amino acid deletion at the C-terminal segment (Supplementary Figure 1) leading to the loss of a polar contact between the N atom of Lys592 in SNRNP200 and the O atom from Leu2333 in PRPF8 (Supplementary Figure 2).

**FIGURE 1:**
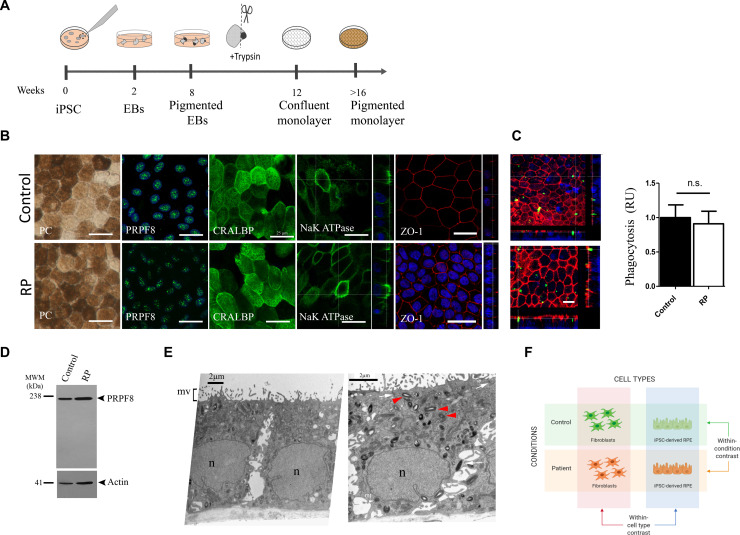
Generation of iPSC-RPE cells from patient and control. **(A)** Schematic of iPSC differentiation toward RPE. **(B)** Characterization of iPSC-RPE. Bright-field images of iPSC-RPE monolayers. Immunocytochemistry to PRPF8 and characteristic RPE markers CRALBP, Na^+^/K^+^-ATPase (Na/K), and ZO-1. Vertical simulation sections for Na^+/^K^+^ ATPase show mainly apical distribution and ZO-1 showing typical apical lateral junction distribution. **(C)** POS phagocytosis assay: FITC-labeled POS (green); F-actin is stained by phalloidin (red) to visualize cell morphology and internalization of POS and quantitative analyses of ingested POS. **(D)** Western blot analysis of PRPF8. **(E)** Transmission electron microscopy of patient iPSC-RPE. Left panel: cuboid polarized RPE cell organized in monolayer with basally located nucleus (n) and apically distributed microvilli (mv). Right panel: RPE cells show melanin granules (red arrowheads) apically from the nucleus, intercellular junctional complexes that include the tight junctions, adherens junctions (arrows), and membrane interdigitations (arrowheads), and basally positioned nucleus (n). **(F)** Contrasts analyzed by RNA-Seq. Created with BioRender (https://biorender.com/). Data are expressed as means ± SD, *n* = 3 assays from two differentiation experiments; Student’s *t* test, n.s., non-significant. Scale bar 25 μm where not indicated.

The iPSCs were derived from the patient and differentiated toward RPE cells according to the previously described procedure (Figure 1A; Hongisto et al., 2017). As a control, a healthy subject’s iPSCs were differentiated toward RPE. The obtained RPE cells exhibited typical native RPE traits including the formation of a cellular monolayer with a polygonal shape and pigmented cells and showed expression of RPE markers such as cellular retinaldehyde-binding protein (CRALBP), sodium/potassium-dependent ATPase (Na^+^/K^+^ ATPase) with predominantly apical distribution, and zonula-occludens-1 (ZO-1), a tight-junction marker, with typical apico-lateral distribution (Figure 1B). PRPF8 exhibited typical nuclear punctate distribution in control and patient’s RPE, with the antibody that recognizes the N- and C-terminal end without any significant difference between two genotypes. The defective protein is 2,328 amino acids long compared to the wild-type, 2,335-amino-acid-long protein. This difference in size could not be detected by Western blot using the antibody raised against the N-terminus (Figure 1D) or the one recognizing the C-terminal amino acids (2,036–2,335) (data not shown). The identity of the iPSC-derived RPE cells was validated using RNA-Seq data, i.e., by DE analysis of several RPE trait markers (BEST1, MERTK, MITF, RLBP1, and RPE65) (Strunnikova et al., 2010) between fibroblasts and RPE cells in each condition (control and patient). Indeed, all these markers are upregulated in the RPE cells in comparison to fibroblasts, with comparable intensity in both control and disease cells (Supplementary Figure 3).

To further confirm the identity of RPE cells, ultrastructural analyses were performed and characteristic polarized RPE morphology was detected in Patient-RPE (Figure 1E) and Control-RPE (data not shown). The polarized organization of cuboidal cells typical of RPE was observed with basally positioned nuclei and apically distributed microvilli (Figure 1E, left panel). Melanosomes represented with black round and oval shapes were present in the apical portion of the cells while ellipsoidal mitochondria were seen below the nuclei, largely displaced toward the basal-lateral part of the cells, the natural position for these organelles *in vivo*. Intercellular junctional complexes are visible in appropriately aligned sections of RPE (Figure 1E, right panel). Again, the patient’s RPE showed the main hallmarks of native RPE without any morphological differences compared to healthy individual control. Furthermore, we assessed the ability of iPSC-RPE to phagocytose POS, mimicking a daily rhythmic binding and internalization of POS tips by RPE cells essential for vision. The ability of patient and control iPSC-RPE was compared by quantifying the FITC-labeled POS fluorescence inside the cells. The results indicate that the disease and control iPSC-RPE ingested the photoreceptor membranes similarly (Figure 1C).

### Functional Analysis of Gene Expression Differences Between Patient and Control Cells

In order to assess the transcriptional changes associated with disease, four pairwise comparisons between samples were performed in order to obtain DE genes between each relevant pair of conditions (Figure 1F and Supplementary Files 1–4). Supplementary Table 1 summarizes the number of DE genes between each pair of conditions. Within-condition contrasts (RPE vs. fibroblasts for either patient or control samples) were performed to reveal differences related to the cell type identity, while the within-cell type contrasts (i.e., comparing patient vs. control for the same cell type) had the potential to unravel differences associated with the disease and the PRPF8 mutation. PCA of the different sample types (Supplementary Figure 4) indicated that cell-type differences (RPE vs. fibroblasts) explained the highest variance across samples (∼50%, PC1), followed by expression changes between the clinical conditions (∼13%, PC2). Nevertheless, the expression profile between healthy and disease RPE cells presented more differences than that of fibroblasts, as expected given that the PRPF8 mutation presents a phenotype only in the retina.

**FIGURE 2:**
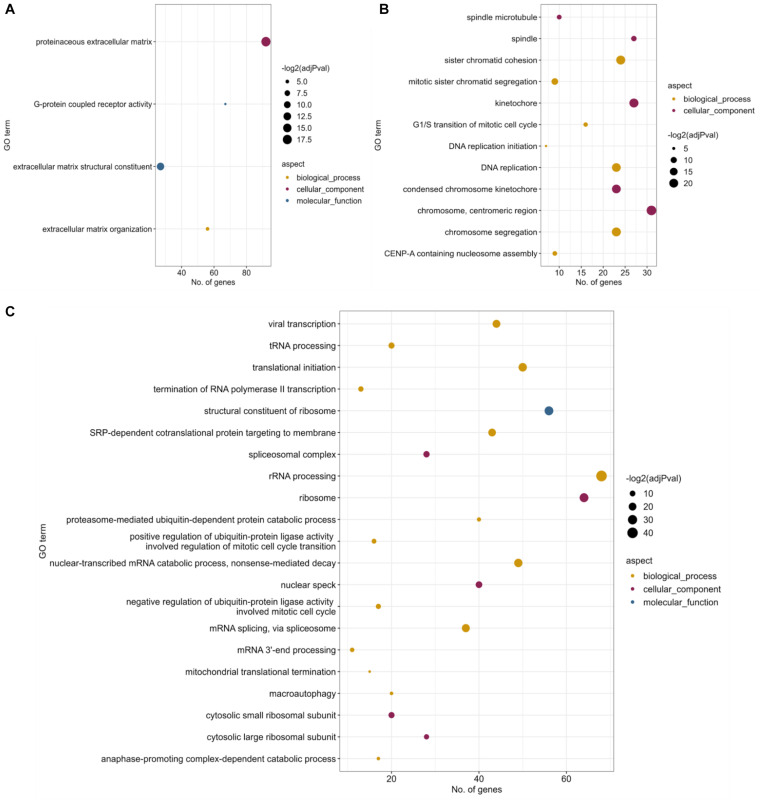
Functional enrichment results for differential gene expression. Significantly enriched (FDR < 0.05) Gene Ontology terms for **(A)** Patient-RPE vs. Control-RPE contrast, filtering genes that are DE between patient and control fibroblasts. **(B)** Patient-RPE vs. Patient-fibroblasts, filtering genes that are DE in the control contrast, and **(C)** Control-RPE vs. Control-fibroblasts, filtering genes that are DE in the disease contrast. Dot size indicates significance of the Fisher’s Exact Test [–log(adj *p* value)], *x*-axis position indicates the number of DE genes that are annotated for each significant GO term, and dot color indicates the GO aspect under which the term is annotated.

For the within-cell type contrasts (Supplementary Files 1, 2), DE analysis of Patient-RPE vs. Control-RPE was performed [False Discovery Rate (FDR)-adjusted *p* value <0.05], subsequently filtering genes that were also DE in Patient-fibroblasts vs. Control-fibroblasts (see *Materials and Methods*). We considered this set of DE genes (4,644, Supplementary Table 1) to be RPE-specific and therefore to encode a relevant part of the transcriptional signature of the disease. Functional enrichment analysis of this set of genes identified significantly enriched (Fisher’s Exact test, FDR < 0.05) GO terms, namely, *G-protein coupled receptor activity* [GO:0004930, 67 genes, 45 (67%) upregulated in patient] and several extracellular matrix-related terms [GO:0005578, GO:0005201, GO:0030198; 92 genes in total, 70 (76%) upregulated in patient] (Figure 2A). This suggests a coordinated increase in expression for these genes in the disease condition. However, some of these genes are depleted in the patient condition and could also contribute to create an imbalance of these functions in the RP disease. Interestingly, none of the 67 genes annotated as G-protein-coupled receptor activity genes were differentially spliced in any of the comparisons performed (see the next section). Therefore, expression dysregulation without splicing alterations could be considered a secondary effect of the PRPF8 mutation. Among 92 genes annotated under an extracellular-matrix-related term, some show a splicing change in the within-cell-type contrasts, namely, *COL5A1*, *FN1*, *LAMA2*, *VEGFA*, and *HAPLN3*. All except *HAPLN3* show differential splicing for at least one event in all contrasts, suggesting a possible direct impact of the mutated splicing factor.

Regarding the two within-condition contrasts (Control-RPE vs. Control-fibroblasts and Patient-RPE vs. Patient-fibroblasts, Supplementary Files 3, 4), we filtered common DE genes to obtain transcriptional signatures of RPE cell-type identity, i.e., healthy and disease RPE phenotypes. We observed 4,903 unique DE genes (*p* < 0.05) for the control comparison (Supplementary Table 1), which represent differences in expression between RPE and fibroblasts required for encoding healthy cell-type identity and presumably lost due to the disease mutation. For the disease condition, 1,838 unique DE genes were identified (*p* < 0.05, Supplementary Table 1), probably related to the development of the disease in RPE cells. The majority of common DE genes (7,491) present a similar change in expression between both cell types, supporting the robustness of the transcriptional differences between RPE and fibroblasts (Supplementary Figure 4).

Functional enrichment analysis of DE genes unique to the patient contrast (Patient-RPE vs. Patient-fibroblasts) identified significant enrichment (Fisher’s Exact test, FDR < 0.05) of a broad set of biological functions related to cell division and the cytoskeletal elements participating in mitosis, including *spindle* (GO:0005819), *chromosome segregation* (GO:0007059), and *kinetochore* (GO:0000776) (Figure 2B). The functional enrichment analysis for DE genes unique to the control contrast (Control-RPE vs. Control-fibroblasts) included a much wider range of significantly overrepresented (Fisher’s Exact Test, FDR < 0.05) biological processes, including mitosis-related GOs, but also terms associated to splicing (*spliceosomal complex*, GO:0005681; *mRNA splicing via spliceosome*, GO:0000398; *mRNA 3*′*-end processing*, GO:0031124; *nonsense-mediated decay*, GO:0000184) and ribosome structure and translation (*ribosomal subunits*, GO:0022625 and GO:0022627*; rRNA processing*, GO:0006364*; translational initiation*, GO:0006413 *SRP-dependent cotranslational targeting of membrane*, GO:0006614) (Figure 2C). Ribosome/translation-related terms included 76 genes, of which 69 (91%) were upregulated in Control-RPE. This coordinated upregulation does not appear in the case of the patient contrast, suggesting depletion of ribosomal functionality in Patient-RPE when compared with fibroblasts of the same clinical condition. Meanwhile, AS-related terms included a total of 45 genes, 26 (58%) of which were downregulated in Control-RPE, showing a relative balance of up-/downregulation of AS genes. In the patient contrast, no coordinated DE or significant overrepresentation was observed and pinpoints to the dysregulatory effect of the PRPF8 mutation on splicing machinery components, which have been shown to undergo widespread regulation by post-transcriptional mechanisms (Lareau et al., 2007; Ni et al., 2007; Han et al., 2010; Anko et al., 2012; Lareau and Brenner, 2015).

### AS Signature of Healthy and Disease RPE Cells

In order to shed light on the mutant PRPF8 pathomechanism, we analyzed splicing patterns in the different cell types and clinical conditions and we evaluated the difference in Percentage Spliced In (dPSI, see section “Materials and Methods”) for each of the four contrasts (Figure 1D and Supplementary Files 5–8), considering the following splicing event categories: alternative 3′ and 5′ site (A3/A5), alternative first (AF) and alternative last (AL) exon, mutually exclusive (MX) exons, retained intron (RI), and skipped exon (SE) (Trincado et al., 2018).

**FIGURE 3:**
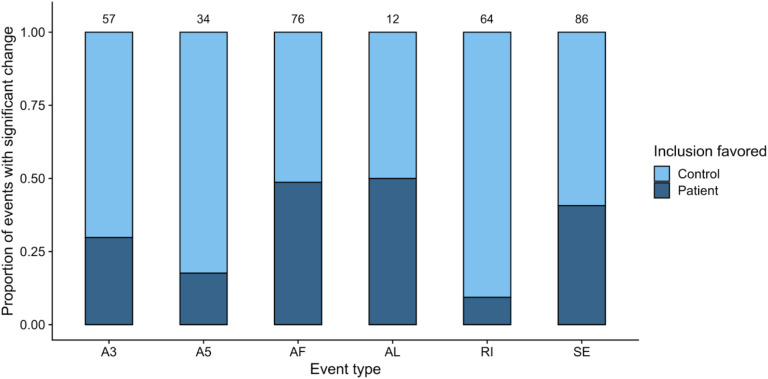
Summary of alternatively spliced events (*p* value <0.05) in the RPE contrast. Only events unique to the Control-RPE vs. Patient-RPE contrast are shown. The proportion of events for which inclusion is favored in each clinical condition is indicated by the bar area. The number of events for each category is shown in the label above the bar. Event types: A3, Alternative 3′ site; A5, Alternative 5′ site; AF, Alternative First exon; AL, Alternative Last exon; MX, Mutually Exclusive Exons; RI, Retained Intron; SE, Skipped Exon.

For the within-cell-type comparisons (Supplementary Files 5, 6), we observed a higher number of splicing events with significant differences (*p* value <0.05) when comparing RPE cells from different clinical conditions than in the case of fibroblasts (Supplementary Figure 5), which we attributed to the high complexity of splicing in RPE (Strunnikova et al., 2010; Farkas et al., 2013) and the fact that fibroblasts show no disease phenotype as a result of the PRPF8 mutation. After removing common significant events from the RPE contrast (Supplementary Table 2), we used dPSI values to check the direction in the splicing change, i.e., the clinical condition in which event inclusion is promoted for RPE cells (Figure 3). A strong tendency toward inclusion of RI events can be observed in Control-RPE in comparison with Patient-RPE, as well as promoted inclusion of A3 and A5 sites, while there were no global direction change patterns in AF, AL, or SE events.

**FIGURE 4:**
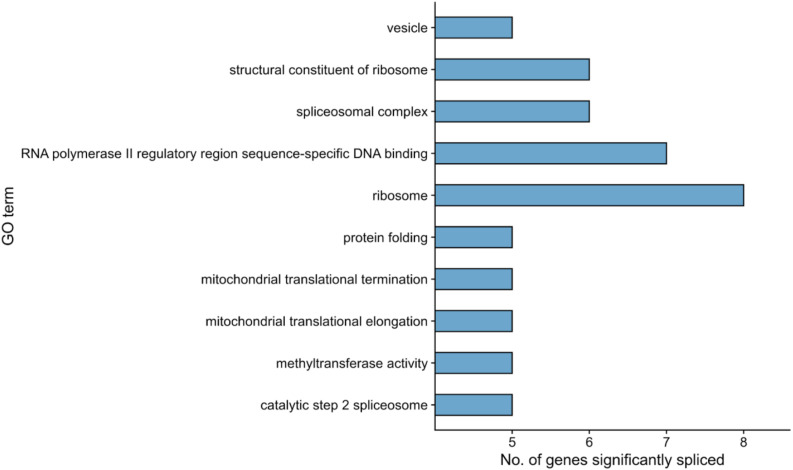
Functional enrichment results for differentially spliced genes in the RPE contrast (Control-RPE vs. Patient-RPE, only unique events). The top-10 most frequently annotated GO terms are shown in the *y*-axis. The number of genes belonging to each GO term is indicated by bar height (*x*-axis).

**FIGURE 5:**
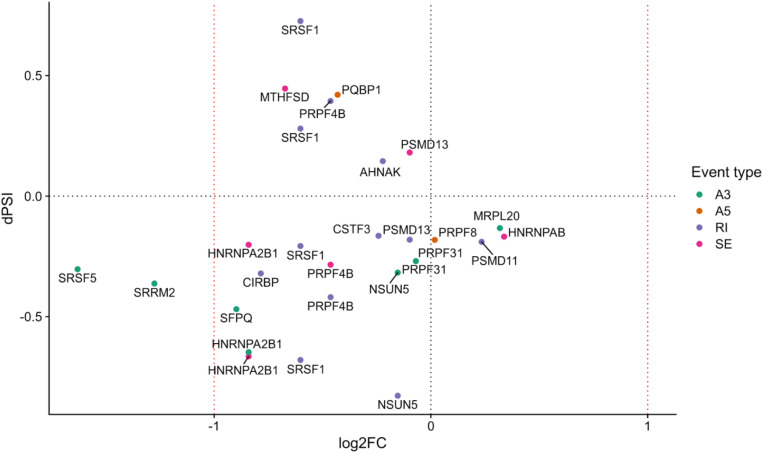
Splicing-related genes with at least one differentially spliced event (*p* value <0.05) in the Control-RPE vs. Patient-RPE contrast (only genes unique to the RPE contrast are considered). Each event is colored by type (A3, alternative 3′ site; A5, alternative 5′ site; RI, retained intron; SE, skipped exon). dPSI is shown in the *y*-axis (dPSI < 0: event inclusion favored in control, bottom panel; dPSI > 0: event inclusion favored in patient, upper panel). log2(Fold Change) is shown in the *x*-axis (FC < 0: gene downregulated in patient, left panel; FC > 0: gene upregulated in patient, right panel).

We next performed a functional enrichment analysis of genes with at least one significant AS event, removing events common between the fibroblast and RPE contrasts as previously described for the DE analysis (see section “Materials and Methods”). Functional analysis of events unique to the RPE contrast showed no significantly overrepresented terms (FDR < 0.05). Hence, we ranked GO terms by their frequency of annotation in alternatively spliced genes to gain functional insight into the splicing patterns of the disease. The most frequently annotated GO terms were *RNA polymerase II DNA binding* (GO:0000977), including several alternatively spliced zinc finger genes (*ZNF154*, *ZNF211*, *ZNF419*, and *ZNF691*), as well as *ribosome* (GO:0005840) and *structural constituent of ribosome* (GO:0003735), which included some genes also annotated for mitochondrial translation, i.e., Mitochondrial Ribosome Protein genes from the Large subunit (MRPL genes). In addition, several splicing-related terms were among the most frequently annotated (Figure 4), namely, *spliceosomal complex* (GO:0005681) and *catalytic step 2 spliceosome* (GO:0071013). Genes under these annotations included *PRPF4*, *PRPF8*, and *PRPF31*, known to be involved in RP (Sullivan et al., 2006; Chen et al., 2014), as well as *SRSF1*, *HRNPA2B1*, and *SRRM2* (Figure 5). This suggests a high level of functional dependence between different spliceosome components that have been shown to cause RP and seems to further indicate that PRPF8 malfunction may propagate to other spliceosome components to generate the disease phenotype. In fact, *SRRM2* and *SRSF1* are also DE in the RPE contrast (downregulated in patient). Therefore, the PRPF8 mutation could have a dual role in altering both the expression and the splicing of other spliceosomal genes and, potentially, their function. Ultimately, we hypothesize that the RP disease would be caused by the cumulative effect of these alterations in the splicing machinery.

In order to better understand the mechanism by which the PRPF8 mutation caused mis-splicing in splicing-related and non-splicing related genes, we analyzed the relationship between gene expression and splicing for each type of event (Supplementary Figures 6–9). Regarding RI events, we observed increased intron retention in Control-RPE that seemed to be decoupled from transcriptional regulation (Supplementary Figure 6), since intron retention does not appear to be related to a pattern of gene downregulation in the control condition. Nevertheless, *SRSF1* and *PRPF4B*, the latter involved in AS, contradicted this pattern and showed highly included IR events in Patient-RPE, together with a decrease in expression in this clinical condition. This deviation from the general pattern for *SRSF1* and *PRPF4B* agrees with previous reports of increased intron retention in RP (Martinez-Contreras et al., 2007) as well as with its established role in decreasing expression levels of transcripts (Braunschweig et al., 2014) and may suggest a direct role for *PRPF8* in the splicing of these two genes, which generates an imbalance in coding vs. non-coding isoforms when the mutation is present.

In the case of SE events, we observed a similar number of patient and control-favored inclusion of alternative exons (Supplementary Figure 7). However, there seems to be a global tendency toward downregulation in patient for this group of genes. Importantly, this category of events includes alternatively spliced HNRNP genes (*HRNPA2B1, HNRNPDL, HNRNPAB*), which are known to participate in splicing control (Martinez-Contreras et al., 2007) as well as the RNA-binding protein MTHFSD, whose deregulation has been previously linked to disease (MacNair et al., 2016).

Regarding A5 and A3 sites, we observed very different behavior as a response to the mutation in PRPF8. While few A5 sites showed a significant splicing change (Supplementary Figure 8), with inclusion of the alternative portion of the exon being favored neither in patient nor in control, our analyses revealed a strong tendency toward A3 site inclusion in the Control-RPE (Supplementary Figure 9). Patient-RPE therefore showed more frequent usage of the A3 sites within these exons, while healthy cells (Control-RPE) tend to use the site that generates a longer exon. These results suggest an interaction between PRPF8 and the 3′ splice site. In addition, these splicing changes do not seem to be related to a DE pattern when comparing patient and control RPE cells (Supplementary Figure 9). Therefore, this PRPF8-mediated global A3 event inclusion, potentially caused by the mutation, would solely be associated to post-transcriptional regulation, in contrast to SE and IR results. AS-related genes showing this pattern included previously discussed *SRSF5, PRPF31, HRNPA2B1*, and *SRRM2*, as well as splicing factor *SFPQ* and RNA methyl-transferase *NSUN5* (Supplementary Figure 9).

### AS Signature Encoding Healthy and Disease Cell-Type Identities of RPE Cells

Aiming to understand the contribution of AS to RPE cell identity, we analyzed within-condition changes in AS events (Supplementary Files 7, 8). We presumed significant AS events and AS genes unique to the control contrast (Control-fibroblasts vs. Control-RPE) to be key determinants of healthy RPE cells that are no longer present in the patient contrast. Meanwhile, unique significant AS events and genes in the patient contrast (Patient-fibroblasts vs. Patient-RPE) constitute splicing alterations that arise due to the disease. This analysis revealed an overall decrease in the number of significant events (*p* value <0.05) in the patient contrast (Supplementary Figure 10), which could be attributed to a depletion in spliceosomal function caused by PRPF8 mutation.

**FIGURE 6:**
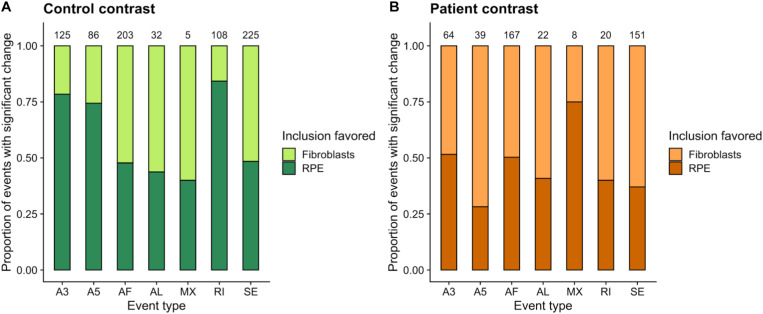
Summary of unique alternatively spliced events (*p* value <0.05) detected in the control **(A**, Control-fibroblasts vs. Control-RPE) and patient **(B**, Patient-fibroblasts vs. Patient-RPE) contrasts. The proportion of events for which inclusion is favored in each clinical condition is indicated by bar area. The number of events for each category is shown in the label above the bar. Event types: A3, Alternative 3′ site; A5, Alternative 5′ site; AF, Alternative First exon; AL, Alternative Last exon; MX, Mutually Exclusive Exons; RI, Retained Intron; SE, Skipped Exon.

**FIGURE 7:**
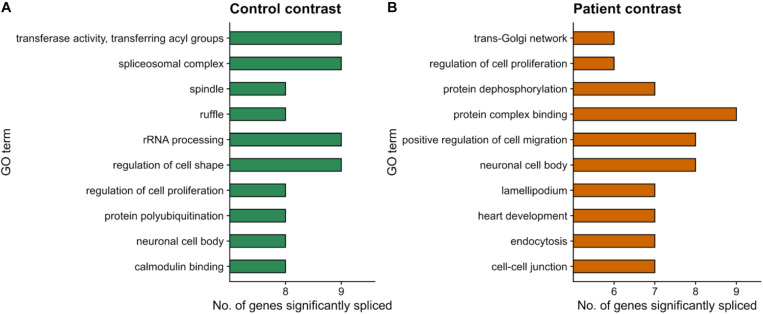
Functional enrichment results for differentially spliced genes (*p* value <0.05) in the within-condition contrasts. **(A)** Control-fibroblasts vs. Control-RPE. **(B)** Patient-fibroblasts vs. Patient-RPE. Only events unique to each contrast are considered. The top-10 most frequently annotated GO terms are shown in the *y*-axis. The number of genes belonging to each GO term is indicated by bar height (*x*-axis).

We next used the dPSI values to look at event inclusion/exclusion patterns between cell types in each clinical condition (Figure 6). Regarding AF and AL events, there were no trends in either clinical condition, or high variation in the number of events with a significant splicing change between control (Figure 6A) and patient (Figure 6B). Interestingly, events in the rest of the categories showed preferential inclusion in one cell type in at least one of the clinical conditions or a drastic change in the number of significant AS events. AF and AL events are generated due to alternative start/end sites for transcription and therefore correspond to transcriptional regulation mechanisms, which further supports the hypothesis that the PRPF8 mutation has its effect via the alteration of post-transcriptional regulation, i.e., splicing. In the case of RI, while intron inclusion was favored in fibroblasts for most RI events in the patient contrast (which matches the decrease in RI detected for Patient-RPE in the within-cell type contrasts), the control contrast revealed a higher degree of intron retention in RPE. Of note, the number of significant RI events was much higher in the control contrast (108) in comparison to patient (20), suggesting the existence of an intron retention program that could be important for the determination of the healthy RPE phenotype. A3 and A5 events also tended to be less included in RPE in the patient contrast in comparison to the control, which revealed coordinated usage of exon alternative sites in Patient-RPE, as could be observed for A3 sites in the RPE contrast. Ultimately, this suggests a change in the way that PRPF8 interacts with both the 3′ and 5′ splice sites in the RP disease. Functional enrichment analysis of unique AS genes showed no significantly overrepresented GO terms (FDR < 0.05) in either contrast. We therefore looked at the frequencies of GO term annotations for these genes (Figure 7). Interestingly, genes whose splicing status encodes the healthy RPE phenotype (control contrast) are frequently annotated for *spliceosomal complex* (GO:0005681, 9 genes) and *rRNA processing* (GO:0006364, 9 genes), two functional terms that appear linked to the PRPF8 mutation and RP throughout this study. These findings are in line with our hypothesis regarding the existence of a healthy splicing signature, involving spliceosome and ribosomal genes, that is required to maintain correct retinal function and is disrupted via splicing defects initiated with defective PRPF8. Another cell function that was shown to be enriched in genes DE in the control contrast (Figure 2), *regulation of cell proliferation* (GO:0042127, eight genes), was the only common GO term among the most frequent annotations.

Finally, we studied the events that change in each within-condition contrast for the genes annotated under these three GO terms (Supplementary Figures 11–15). Regarding *rRNA processing* genes (Supplementary Figure 11), we observed variable trends regarding the cell type in which inclusion of the AS events is favored. While *PLXNB3*, *CFAP410*, and *RHOJ* presented SE events that showed inclusion in fibroblasts, a similar event in *MKLN1* was included in RPE. Regarding event categories, although several genes showed differentially spliced AF exons, we did not observe a pattern among significant event types for rRNA processing genes. However, FN1 and RASA1 present A3 and A5 sites, respectively, showing inclusion in RPE, matching the previously described trend (Figure 6). For spliceosome genes (Supplementary Figure 12), however, we noticed that most significant events were IR, A3 and A5 sites, while very few events within these three categories were observed in rRNA processing genes (Supplementary Figure 11). The abundance of A3 and A5 events changing among splicing-related genes could further support our hypothesis that WT PRPF8 is involved in spliceosomal binding to the splice sites. Indeed, all A3 and A5 sites showed inclusion in RPE (Supplementary Figure 12) and therefore selection of the splice sites that generated longer exons. This splicing change is lost in the patient contrast, suggesting a lack of control over this process in the disease for these set of spliceosomal genes, which include helicase *DHX37*, ribosomal components *RPL15* and *RPL31*, and several ribosome maturation factors and rRNA processing proteins. The exact role of splice site selection in the RP disease, however, requires further validation and is beyond the scope of this study.

Genes annotated for *regulation of cell proliferation* were the only functional group that was frequently annotated among AS genes in both clinical conditions, although the sets of genes in each contrast are different, with the exception of *NF2*. In the case of this gene group, most splicing changes occurred in SE and AF events, except for an A5 event detected in the control contrast (*STAT6*) and an A3 event in patient (*ITSN1*). We consider these, together with genes presenting SE events (control: *CNN2*, *IL4R*, *PKD2*, and *PIAS1*; patient: *MYH10, PALLD*, and *WASF2*), to be genes subject to strong post-transcriptional regulation and therefore candidates for future evaluation of the impact of these splicing changes on RPE function. Finally, among the 371 common events (significant in both contrasts), we found 14 events that presented different dPSI sign in the patient and control comparisons (Supplementary Figure 15), i.e., alternatively spliced events with different direction of change depending on the clinical phenotype. In addition to transcriptionally controlled AF exons, common events consisted mainly of SE that presented preferential inclusion in RPE in the control contrast (*MRPS31, MTX1, SHLD2P3*, and *HNRNPDL*), while the exon becomes excluded in RPE in the patient contrast. This set of genes included *HNRNPDL*, associated with pre-mRNA processing. A similar trend is observed for A3 events in the *CYB5D1* and *TPM2* genes, as well as for an RI event in the SNORD locus (C/D box Small Nucleolar RNAs).

In summary, after performing within-condition contrasts, we have observed a pattern of event inclusion in the control contrast that is lost in patient, when not completely reversed, as is the case of common genes. This mainly affects SE, A3, and A5 events for genes involved in splicing, rRNA processing, and proliferation. The role that the skipped exons or exon fragments play in the gene’s functionality is to be defined; however, our analyses suggest that the PRPF8 mutation creates this event exclusion pattern. We believe that these changes could alter the splicing signature required for healthy RPE identity and that they may be a consequence of AS dysregulation in the RP disease. Ultimately, this list of alternatively spliced events and genes should constitute a valuable resource for further validation in order to find candidates that can explain the PRPF8-mediated RP pathomechanism.

## Discussion

Despite huge progress in the identification of genetic causes of the disease, the pathomechanism behind RP is largely unknown. While animal models have proven useful in many cases, albeit in the case of splicing factor-induced RP, they remain insufficient as the disease phenotype is poorly manifested. Moreover, the wide mutational landscape of splicing factor-caused RP is time- and cost-consuming to capture in animal models. In addition, studying disease-causing variants in genetic backgrounds that are demonstrably permissive of the disease is highly desirable in the case of splicing factor-induced RP due to high clinical diversity.

We set out to explore the transcriptional landscape of patient non-affected and affected cells in order to elucidate the unique role of PRPF8 in its target cells, namely, RPE cells vs. non-retinal tissue. The use of iPSCs that can be differentiated toward any human tissue leverages the lack of access to native retinal tissue.

After differentiation to RPE, patient and control cells were indistinguishable upon assessment of morphological, immunoreactive markers. We did not observe basal deposits or infoldings as described in the mouse model (Graziotto et al., 2011). PRPF8 immunofluorescence was equivalent in both genotypes, likely as a result of mutant protein escape from nonsense-mediated decay due to the short C-terminal amino acid sequence loss. Additionally, after assessing POS phagocytosis capacity, we found that patient and control cells behave indistinctly. The difference with the *Prpf8*^*H2309P*/*H2309P*^ mice, which showed reduced phagocytosis, possibly lies in the homozygous state of the causative mutation in the rodent model. The lack of morphological or protein marker expression differences between control and RP genotypes probably reflects the late-onset disease phenotype that emerges as a result of additional cumulative molecular events. It has been previously described that modeling late-onset diseases requires additional cellular insults, since reprogramming leads to erasure of age markers in the original fibroblasts (Horvath, 2013; Miller et al., 2013). Similar results were reported by Foltz et al. with PRPF8 (c.6901C > T, p.P2301S) mutant, who describe iPSC-RPE with similar apicobasal polarity and phagocytosis rate between the patient and gene-corrected isogenic line (Foltz et al., 2018). The rationale behind using the conventional differentiation paradigm toward disease-specific cell type in this work is that mutations in ubiquitously present splicing factors involved in a fundamental cellular process such as splicing is expected to capture early cell-type-specific events prior to overt morphological or functional degeneration.

We then examined mRNA expression and splicing patterns in patient and control fibroblasts and iPSC-derived RPE cells in order to decipher the tissue-specific pathomechanism of the disease. Differential expression analyses between control and patient RPE cell types identified G-protein coupled receptor (GPCR) activity genes and extracellular matrix-related genes, some of the latter also showing changes in AS. GPCRs, the largest family of membrane proteins, act through ligand binding and are responsible for a wide variety of physiological roles. Extracellular matrix proteins are known to play a crucial role in supporting RPE cells *in vivo* and in vitro (Sorkio et al., 2014). RPE synthesizes components of extracellular matrix proteins of Bruch’s membrane and interphotoreceptor matrix, both of which are essential for RPE integrity and function. We hypothesize that these genes could be directly involved in the disease mechanism and constitute good candidates for further validation.

Within-condition differential expression analysis (fibroblasts vs. RPE cells in healthy and disease condition) identified a set of common DE genes associated with mitosis-related processes and components. Importantly, control contrast-unique DE genes showed significant enrichment of splicing and ribosome structure and translation terms, while this signature was completely lost in the patient contrast. This suggests that splicing and ribosome-related processes could be important for cellular identity specification and that their expression dynamics is disrupted in PRPF8-caused RP. In this study, we propose two hypothesis that could explain this mechanism. First, the alterations in the expression of splicing-associated elements could propagate to ribosomal components and translation regulators that may require intervention of the spliceosome for their normal expression. Alternatively, RPE cells may display translational control mechanisms for sustaining RPE properties.

Out of 90% AS genes in humans showing AS, it is estimated that 30% have tissue-specific isoforms (Xu et al., 2002). We set out to identify RPE-specific AS patterns and assess whether these tissue-specific splicing events were affected by the disease. To globally profile AS, we analyzed five modes of splicing events, all of which allow for a single gene to encode multiple protein variants that can have different functional properties. Among the within-cell-type contrasts, a higher number of significant splicing changes were detected when analyzing AS events in RPE cells in comparison to fibroblasts, consistent with the higher functional complexity of RPE cells and the primary effect of the PRPF8 mutation on these cells. Retained introns, as well as alternative 3′ and 5′ sites, showed favored inclusion in Control-RPE compared to Patient-RPE. Intron retention is classically considered as a result of mis-splicing and regarded as a mechanism of suppression of inappropriate transcripts presumably destined for nonsense-mediated decay (Braunschweig et al., 2014). However, emerging studies have demonstrated that introns are actively retained in mature mRNA and may have specific roles in normal physiology (Wong et al., 2016; Adusumalli et al., 2019). Some of the most significantly differentially included intron retention events belonged to genes annotated for splicing and ribosome-related GO terms. These included splicing factors such as PRPF4, PRPF8, and PRPF31, all of which are known to cause RP. Of note, SRSF1 and PRPF4B were highly included in patient-RPE and also downregulated in this condition.

For alternative first and last exons, which are equivalent to Alternative Transcription Start and End Sites, inclusion was not globally favored in any of the conditions. This is not surprising, given that transcription start and end site choice is driven by the transcriptional machinery, instead of by the spliceosome, which operates after transcription is initiated, either post-transcriptionally or simultaneously to transcription (Herzel et al., 2017). The lack of global direction changes could indicate that the disease phenotype is caused by the PRPF8 mutation and that it directly involves splicing rather than transcriptional alterations. Ultimately, usage of AL or AF sites could be stochastic or a functional consequence of splicing alterations in certain proteins, but hardly the root of the disease.

In sum, we identified 27 splicing-related genes, including PRPF8, with at least one differentially spliced event between Control-RPE and Patient-RPE. This result opens interesting possibilities regarding the role of PRPF8 as a splicing factor, regarding both autoregulation of AS of its own pre-mRNA and that of 26 additional splicing factors. In the context of the RP disease, PRPF8 may act as a master AS regulator in RPE cells, targeting a broad set of splicing-involved genes, leading to the propagation of defective PRPF8 function to the rest of the splicing machinery via splicing dysregulation. Similar results have already been described in literature, where the core splicing proteins were found to regulate RNA processing and RNA-binding factors (Saltzman et al., 2011). We hypothesize that these are suitable candidate genes and events to be further validated as RP disease markers and are key to explain how PRPF8-initiated splicing defects propagate to the entire splicing machinery.

Within-condition contrasts showed a higher number of significant AS changes in control over disease cells, similar to the DE results, indicating a depletion of spliceosomal function by PRPF8 mutation. For instance, there were 108 significantly changing intron retention events in the control vs. 20 in the disease contrast. In addition, functional enrichment results for control contrast AS genes identified splicing and ribosome-related functions within the top 10 most frequently annotated GO terms. These within-condition AS results corroborate splicing machinery disruption hypothesis as a potential mechanism for PRPF8-induced RP. Meanwhile, the only common term between control and disease cell types was regulation of cell proliferation, although different genes were identified as AS in each condition’s contrast. This finding is in line with previous reports where PRPF8 was found to affect mitotic cell cycle and RNA splicing GO terms after its depletion by siRNA (Wickramasinghe et al., 2015). After removing genes common to both contrasts, we identified sets of AS-related genes in the control but not in the patient contrast, which were involved in spliceosome machinery, ribosomal structure, and translation, according to GO annotations. The loss of these splicing patterns when comparing disease cells is an additional evidence of the propagation of alterations in the expression and splicing of spliceosomal components to ribosomal proteins and translation regulators, which may require intervention of the spliceosome for their normal expression. Alternatively, and in line with the second proposed hypothesis, RPE cells may display unique human translational control mechanisms for sustaining RPE properties, which are disrupted due to the pathomechanism of mutant PRPF8. However, either of these hypotheses would require further experimental validation.

AS has been previously described to be affected in the PRPF8-depleted condition by siRNA in established cell lines (Tanackovic et al., 2011). Although these conditions do not capture the physiological cell environment and co-expression of the wild-type protein, the data support the role of splicing defects in PRPF8 pathomechanism. Similarly, in a comprehensive PRPF31 study using iPSC-derived RPE cells, differential exon usage was found to affect splicing process itself (Buskin et al., 2018). Moreover, AS GO term analysis of mouse *Prpf*31^+ ⁣/−^ retina and RPE were enriched for genes involved in mRNA processing. The iPSC-RPE from the PRPF31-affected patients exhibited a range of cellular defects, including loss of apico-basal polarity, altered pigment epithelium-derived factor (PEDF), and basal vascular endothelial growth factor (VEGF) secretion, barrier function, phagocytosis, and primary cilia deficiencies. These data are supportive of disrupted AS as a trigger of splicing deficiencies underlying specific biological processes. The observed splicing differences in our iPSC-RPE bearing a mutant PRPF8 copy points to a common primary pathogenic event. The fact that we did not observe morphological and functional disruptions in studied cells can be attributed to a unique PRPF8 mechanism. Another possibility is that our iPSC-RPE were insufficiently matured, which delayed the concomitant cellular events. In that regard, efforts for quantitative quality control to assess maturity instead of image-based analyses are emerging (Plaza Reyes et al., 2020). Parallel studies with both genetic backgrounds are warranted in order to delineate the specific roles of each PRPF gene.

Of note, our study is limited by the small sample sizes in our experimental design. Therefore, further studies involving a larger number of patients would be required to confirm the findings described herein. It is also imperative to determine if different mutations of PRPF8 have a similar impact on the transcriptional profile. As an additional shortcoming, we are aware that the poly(A)-selected RNA profiling described here precludes the involvement of other biologically relevant RNA species, such as microRNAs or long non-coding RNAs, which may also play a role in PRPF8-induced RP (Zhang et al., 2014).

The development of therapies for autosomal dominant retinal diseases with gain-of-function mutations involve strategies to inhibit expression of mutant protein and reduce the ratio of malfunctioned to wild-type protein. Along these lines, DNA oligonucleotides complementary to the pathogenic mRNA (Murray et al., 2015) or gene editing by CRISPR/Cas system targeting mutant allele (Bakondi et al., 2016) were described. These patients would also benefit from gene-independent replacement therapies such as heterologous stem cell replacement or combined genetic and stem cell approach using autologous cells. Importantly, exploring therapies for highly conserved proteins such as PRPF8 can harness the large progeny and short lifespan of lower organisms that allows high-throughput drug or genetic screening (Kukhtar et al., 2020). In view of our findings, the mutated PRPF8 allele-specific therapeutics would be the most effective approach. This, strategy, however, would be efficient if applied very early in the disease before other affected splicing proteins compromise the main cellular functions.

## Data Availability Statement

The datasets presented in this study can be found in online repositories. The names of the repository/repositories and access-ion number(s) can be found below: NCBI, GEO GSE165322.

## Ethics Statement

The studies involving human participants were reviewed and approved by CAEC Valencia. The patients/participants provided their written informed consent to participate in this study.

## Author Contributions

ÁA-L: designed analysis pipeline, performed data analysis, interpreted results, and wrote the manuscript. HS: iPSC expansion and RPE production. AB-A: performed experiments and data analyses. VL: mutation analyses and interpretation. NC: TEM analyses. BD: clinical data. IH, BP, and MC: provided patient’s biopsy. JC, AB, and AD: library preparation and initial data analyses. SE: financial support. DL: overall concept and design, performed the experiments, manuscript writing, and financial support. All authors reviewed the manuscript.

## Conflict of Interest

The authors declare that the research was conducted in the absence of any commercial or financial relationships that could be construed as a potential conflict of interest.

## Footnotes

1 http://www.sph.uth.tmc.edu/retnet

2 http://www.bioinformatics.babraham.ac.uk/projects/fastqc/
