# Supplementary material for: Mutant PRPF8 Causes Widespread Splicing Changes in Spliceosome Components in Retinitis Pigmentosa Patient iPSC-Derived RPE Cells
Source: Front Neurosci. 2021 Apr 29;15:636969. doi: 10.3389/fnins.2021.636969 (PMC8116631; doi:10.3389/fnins.2021.636969)
Supplement: Supplementary file 1 [file Data_Sheet_1.zip › Supplemental_Information/Supplemental Information.pdf]

Mutant PRPF8 causes widespread splicing changes in spliceosome components in retinitis pigmentosa patient iPSC-derived RPE cells

Ángeles Arzalluz-Luque, Jose Luis Cabrera, Heli Skottman, Alberto Benguria, Arantxa Bolinches-Amorós, Nicolás Cuenca, Vincenzo Lupo, Ana Dopazo, Sonia Tarazona, Bárbara Delás, Miguel Carballo, Beatriz Pascual, Imma Hernan, Slaven Erceg and Dunja Lukovic*

SUPPLEMENTAL INFORMATION

Supplementary figures

Supplementary Figure 1. PRPF8 mutation information, gene organization and protein domains.

Supplementary Figure 2. 3D structural representation of SNRNP200 and C-terminus of PRPF8 interaction.

Supplementary Figure 3. RPE marker gene expression in Patient and Control RNA-Seq data.

Supplementary Figure 4. Principal Component Analysis (PCA) of RNA-Seq data by clinical condition and cell type.

Supplementary Figure 5. Summary of significant alternatively spliced events detected in the within-cell type contrasts.

Supplementary Figure 6. Significant intron retention events in the cell type contrast.

Supplementary Figure 7. Significant exon skipping events in the cell type contrast.

Supplementary Figure 8. Significant alternative 5' site events in the cell type contrast.

Supplementary Figure 9. Significant alternative 3' site events in the cell type contrast.

Supplementary Figure 10. Summary of significant alternatively spliced events detected in the within-condition contrasts.

Supplementary Figure 11. Event state summary of unique differentially spliced genes in the control contrast (Control-RPE and Control-fibroblasts) annotated for *rRNA processing* (GO:0006364).

Supplementary Figure 12. Event state summary of unique differentially spliced genes in the control contrast (Control-RPE and Control-fibroblasts) annotated for *spliceosomal complex* (GO:0005681).

Supplementary Figure 13. Event state summary of unique differentially spliced genes in the control contrast (Control-RPE and Control-fibroblasts) annotated for *regulation of cell proliferation* (GO:0042127).

Supplementary Figure 14. Event state summary of unique differentially spliced genes in the patient contrast (Patient-RPE and Patient-fibroblasts) annotated for *regulation of cell proliferation* (GO:0042127).

Supplementary Figure 15. Event state summary of differentially spliced genes common to the patient and control contrasts.

Supplementary tables

Supplementary Table 1. Differential Expression (DE) analysis results for all contrasts.

Supplementary Table 2. Supplementary Table S2. Alternative Splicing (AS) analysis results: summary for within-cell type contrasts.

Supplementary Table 3. Supplementary Table S2. Alternative Splicing (AS) analysis results: summary for within-condition contrasts.

Supplementary Table. 4. Patient's and control information.

Supplementary Files 1-9 (Title and captions)

Supplementary File 1: Differental Expression results for Control RPE vs Patient RPE (within-cell type, RPE contrast), generated using NOISeq. Results included for all analyzed genes (total: 23841). Fold Change (log2FC) < 0 indicates gene downregulated in patient; log2FC > 0: gene upregulated in patient. Significance (FDR) is equivalent to (1 - prob).

Supplementary File 2: Differental Expression results for Control Fibroblasts vs Patient Fibroblasts (within-cell type, fibroblast contrast), generated using NOISeq. Results included for all analyzed genes (total: 23841). Fold Change (log2FC) < 0 indicates gene downregulated in patient; log2FC > 0: gene upregulated in patient. Significance (FDR) is equivalent to (1 - prob).

Supplementary File 3: Differental Expression results for Patient RPE vs Patient Fibroblasts (within-condition, patient contrast), generated using NOISeq. Results included for all analyzed genes (total: 23841). Fold Change (log2FC) < 0 indicates gene downregulated in fibroblasts; log2FC > 0: gene upregulated in fibroblasts. Significance (FDR) is equivalent to (1 - prob).

Supplementary File 4: Differental Expression results for Control RPE vs Control Fibroblasts (within-condition, control contrast), generated using NOISeq. Results included for all analyzed genes (total: 23841). Fold Change (log2FC) < 0 indicates gene downregulated in fibroblasts; log2FC > 0: gene upregulated in fibroblasts. Significance (FDR) is equivalent to (1 - prob).

Supplementary File 5: Differential Alternative Splicing results for Control RPE vs Patient RPE (within-cell type, RPE contrast), generated using SUPPA2. Results correspond to SUPPA2's diffSplice output file. Results shown for significant events only (p-value < 0.05). dPSI < 0: event inclusion favored in control; dPSI > 0: event inclusion favored in patient.

Supplementary File 6: Differential Alternative Splicing results for Control Fibroblasts vs Patient Fibroblasts (within-cell type, fibroblast contrast), generated using SUPPA2. Results correspond to SUPPA2's diffSplice output file. Results shown for significant events only (p-value < 0.05). dPSI < 0: event inclusion favored in control; dPSI > 0: event inclusion favored in patient.

Supplementary File 7: Differential Alternative Splicing results for Patient RPE vs Patient Fibroblasts (within-condition, patient contrast), generated using SUPPA2. Results correspond to SUPPA2's diffSplice output file. Results shown for significant events only (p-value < 0.05). dPSI < 0: event inclusion favored in fibroblasts; dPSI > 0: event inclusion favored in RPE.

Supplementary File 8: Differential Alternative Splicing results for Control RPE vs Control Fibroblasts (within-condition, control contrast), generated using SUPPA2. Results correspond to SUPPA2's diffSplice output file. Results shown for significant events only (p-value < 0.05). dPSI < 0: event inclusion favored in fibroblasts; dPSI > 0: event inclusion favored in RPE.

Supplementary File 9: list of Gene Ontology terms considered as Alternative Splicing-related in functional enrichment analyses.

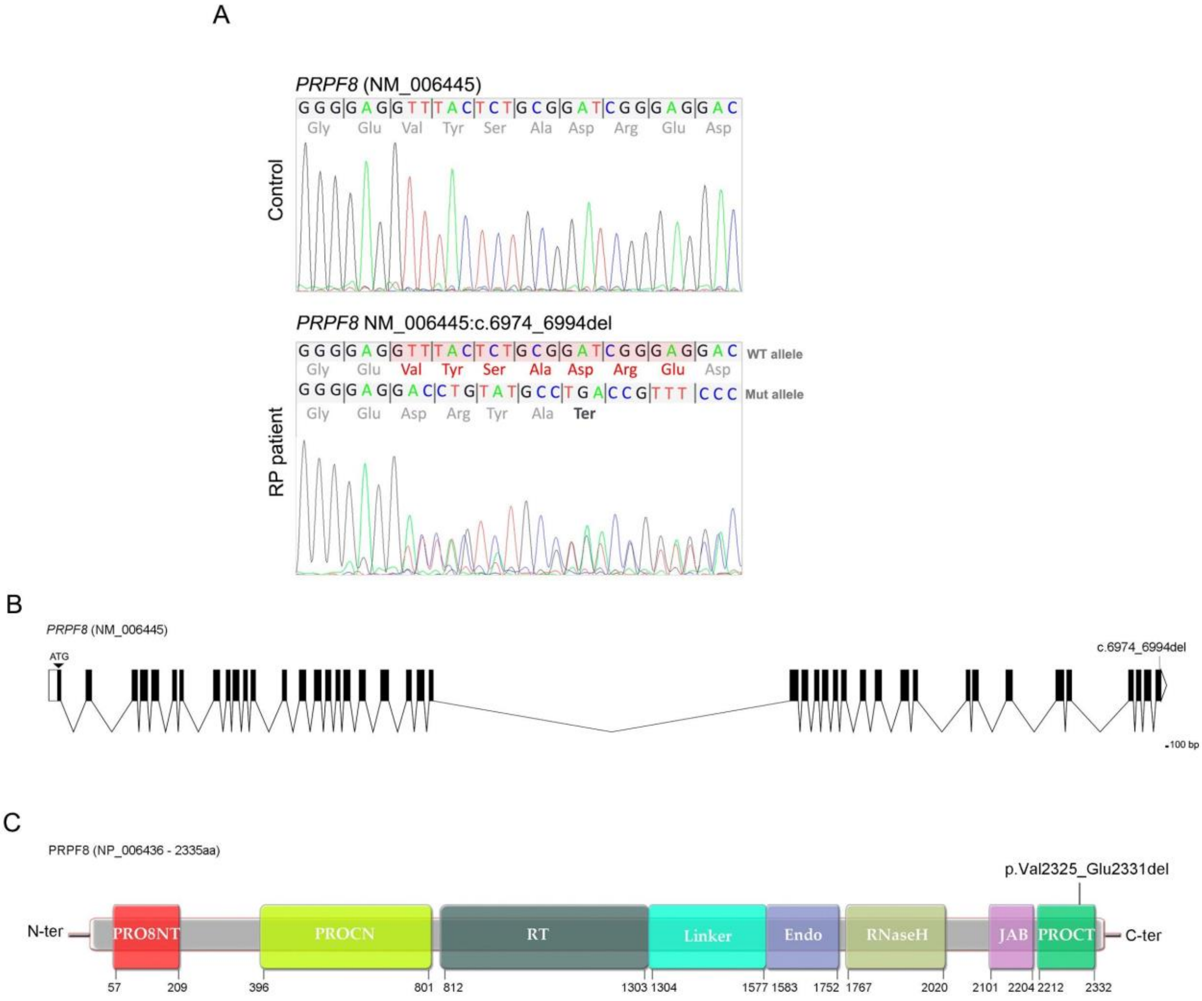

Supplementary Figure 1. PRPF8 mutation information, gene organization and protein domains.

(A) Sequencing chromatograms from unaffected control and RP patient showing the *PRPF8* NM_006445:c.6974_6994del mutation in heterozygous state in the RP patient. Pink shadow indicates the nucleotides deleted in the mutant allele. In bold red are shown the amino acids lost in the mutant allele. **(B)** Schematic diagram showing all exons (black rectangles), introns (black lines) and UTRs (white rectangles) of the PRPF8 gene (NM_006445). The NM_006445:c.6974_6994del variant is located in the exon 42. Scale bar in length= 100 bp. **(C)** Schematic representation of the different domains of PRPF8 protein according to protein database (http://www.rcsb.org/pdb/protein/Q6P2Q9) and location of the NP_006436:p.Val2325_Glu2331del mutation. PRO8NT= PrP8 N-terminal domain; PROCN =central N-terminal domain in pre-mRNA splicing factors of PRO8 family; RT=reverse transcriptase homology domain; Endo=restriction endonuclease homology domain; RNase H=ribonuclease H homology domain; JAB=JAB1/Mov34/MPN/PAD-1 ubiquitin protease, PROCT=C-terminal domain in pre-mRNA splicing factors of PRO8 family.

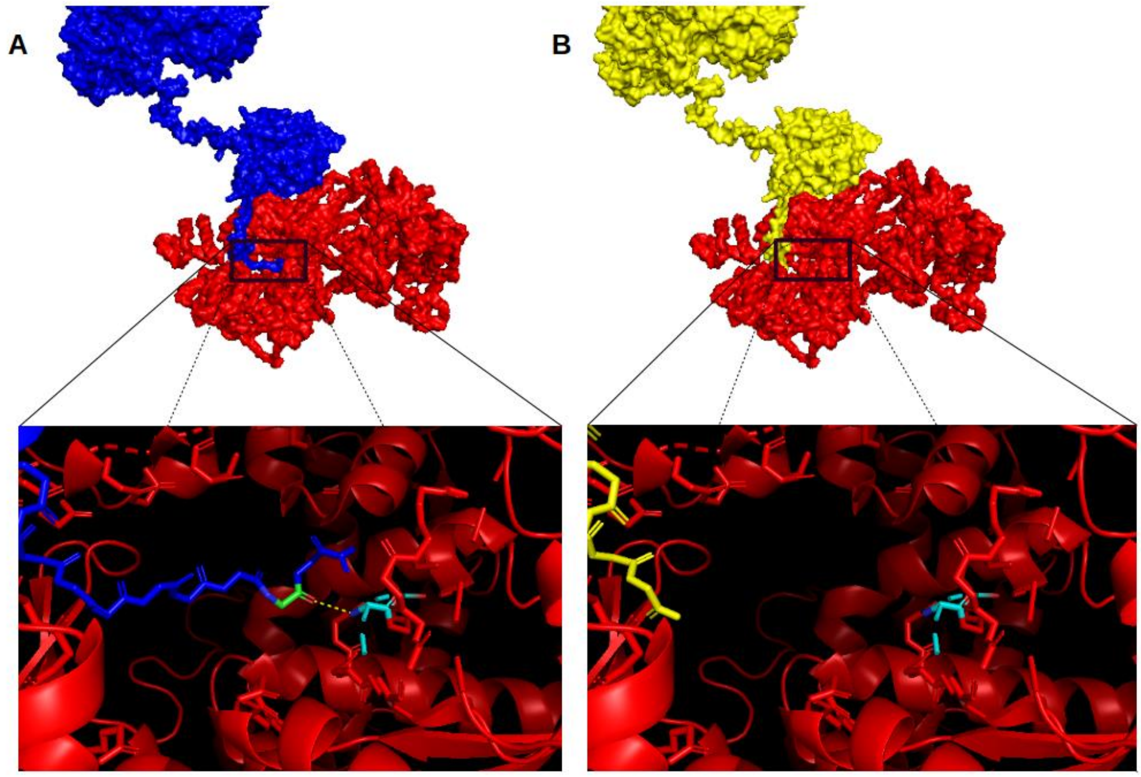

Supplementary Figure 2. 3D structural representation of SNRNP200 and C-terminus of PRPF8 interaction.

A) WT PRPF8 (blue) and SNRNP200 (red). Lys 529 in snRNP200 is depicted in magenta color, Leu 2333 in PRPF8 (green) and polar interaction is represented as dashed yellow line between these residues. B) PRPF8 (p.Val2325_Glu2331del) (yellow) and SNRNP200 (red).

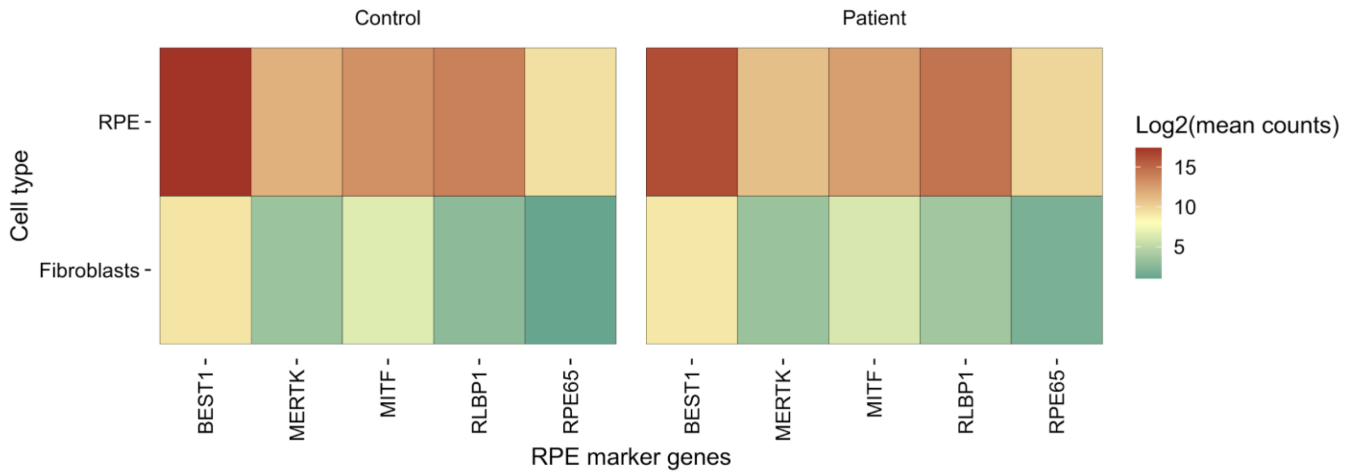

Supplementary Figure 3. RPE marker gene expression in Patient and Control RNA-Seq data.

Heatmap showing the log2(mean expression) of RPE marker genes in Control and Patient cell types. Mean expression was calculated across the three replicates for each of the cell types, in both clinical conditions.

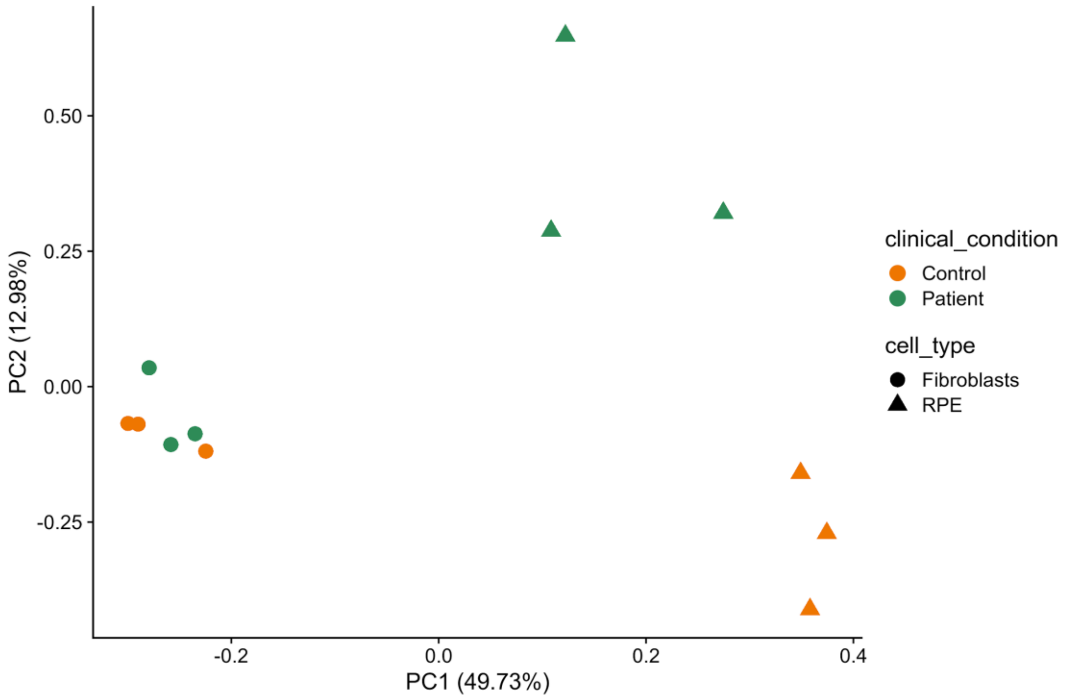

Supplementary Figure 4. Principal Component Analysis (PCA) of RNA-Seq data by clinical condition and cell type.

PCA plot of TMM-normalized and batch-corrected (feature-filtered, 23841 genes) RNA-Seq samples for RPE and fibroblasts from Control and Patient clinical conditions. PC1 and PC2, along with the percentage of explained variability, are represented.

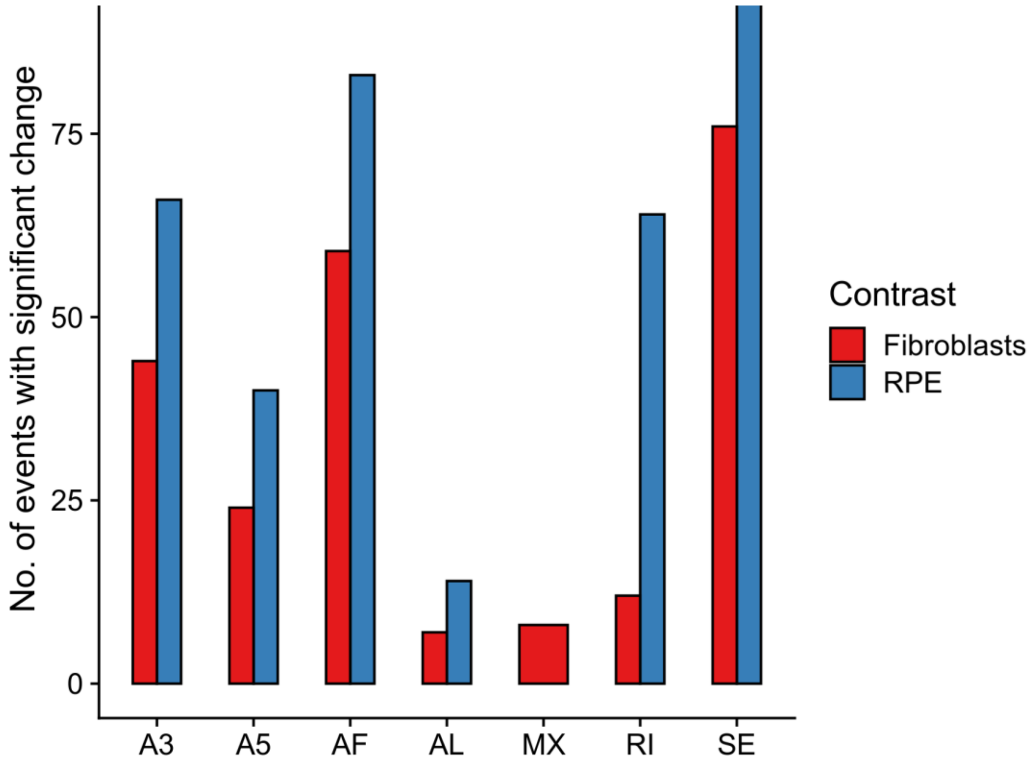

Supplementary Figure 5. Summary of significant alternatively spliced events detected in the within-cell type contrasts.
The y-axis shows the number of significant splicing events ($p<0.05$) for each event category (x-axis), for the within-cell type contrasts, i.e. fibroblast contrast (Control-fibroblasts vs Patient-fibroblasts) and RPE contrast (Control-RPE vs Patient-RPE). Event types: A3: Alternative 3' site; A5: Alternative 5' site; AF: Alternative Final exon; AL: Alternative Last exon; MX: Mutually-Exclusive Exons; RI: Retained Intron; SE: Skipped Exon.

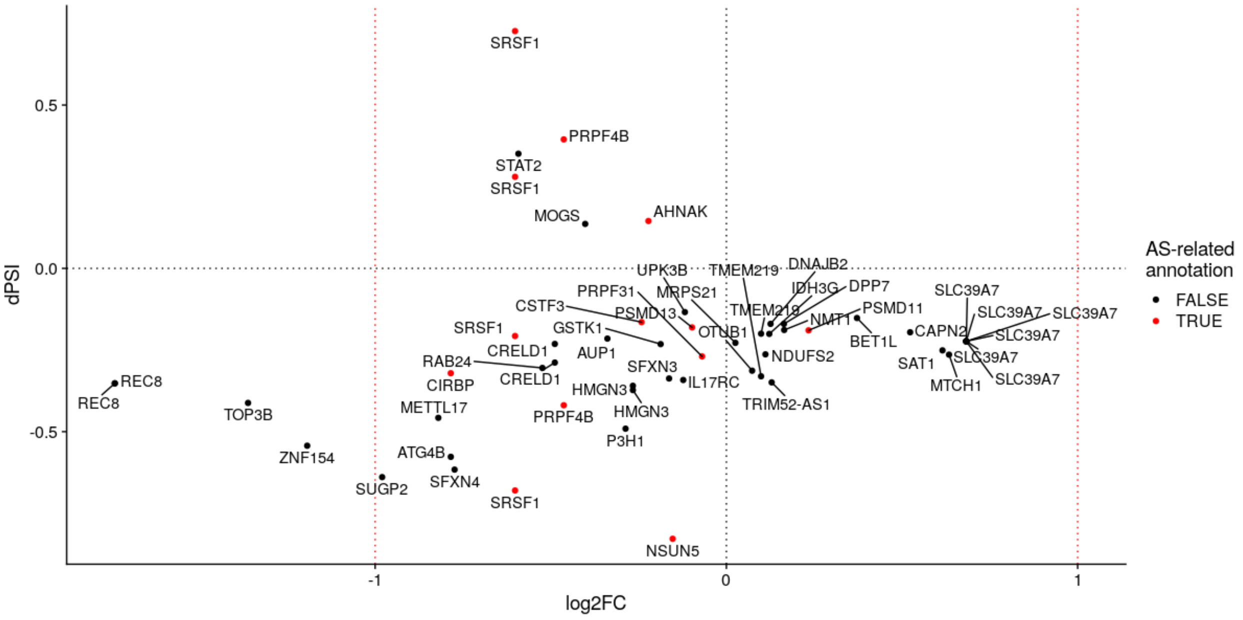

Supplementary Figure 6. Significant intron retention events in the cell type contrast. Significantly AS intron retention events are found in 48 genes. Comparison of dPSI (dPSI < 0 indicates event inclusion favored in Control, bottom panel; dPSI > 0 indicates event inclusion favored in Patient, upper panel) vs log2(Fold Change) (FC < 0: gene downregulated in patient, left panel; FC > 0: gene upregulated in patient, right panel).

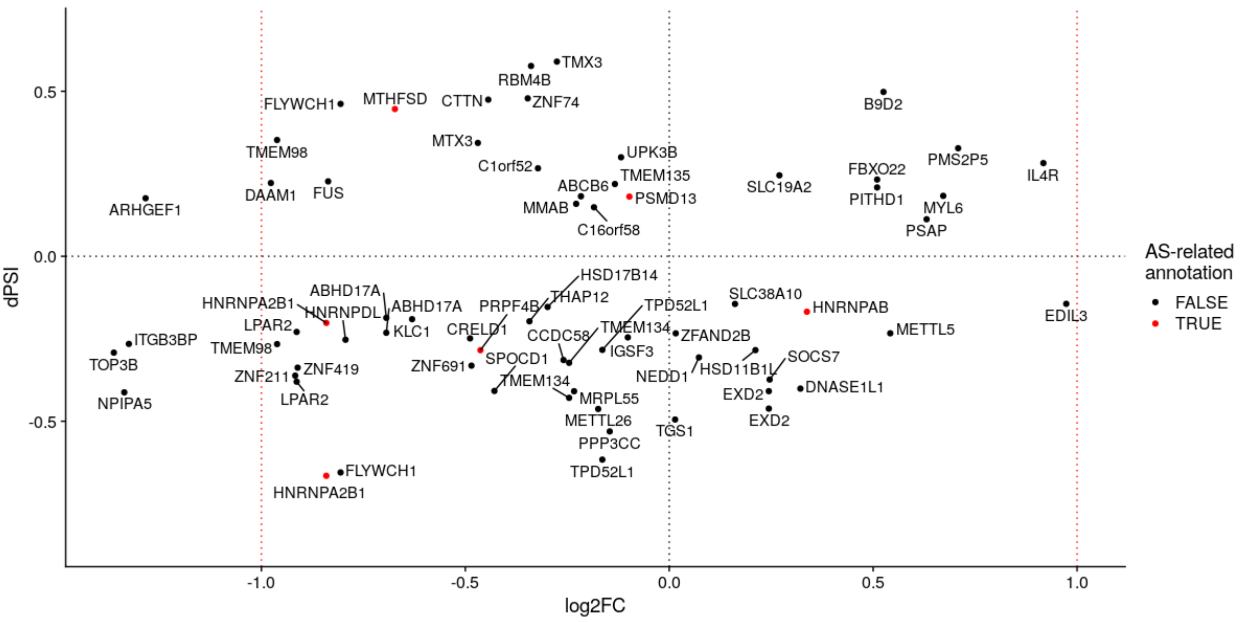

Supplementary Figure 7. Significant exon skipping events in the cell type contrast. Significantly AS exon skipping events are found in 77 genes. Comparison of dPSI (dPSI < 0 indicates event inclusion favored in Control, bottom panel; dPSI > 0 indicates event inclusion favored in Patient, upper panel) vs log2(Fold Change) (FC < 0: gene downregulated in patient, left panel; FC > 0: gene upregulated in patient, right panel).

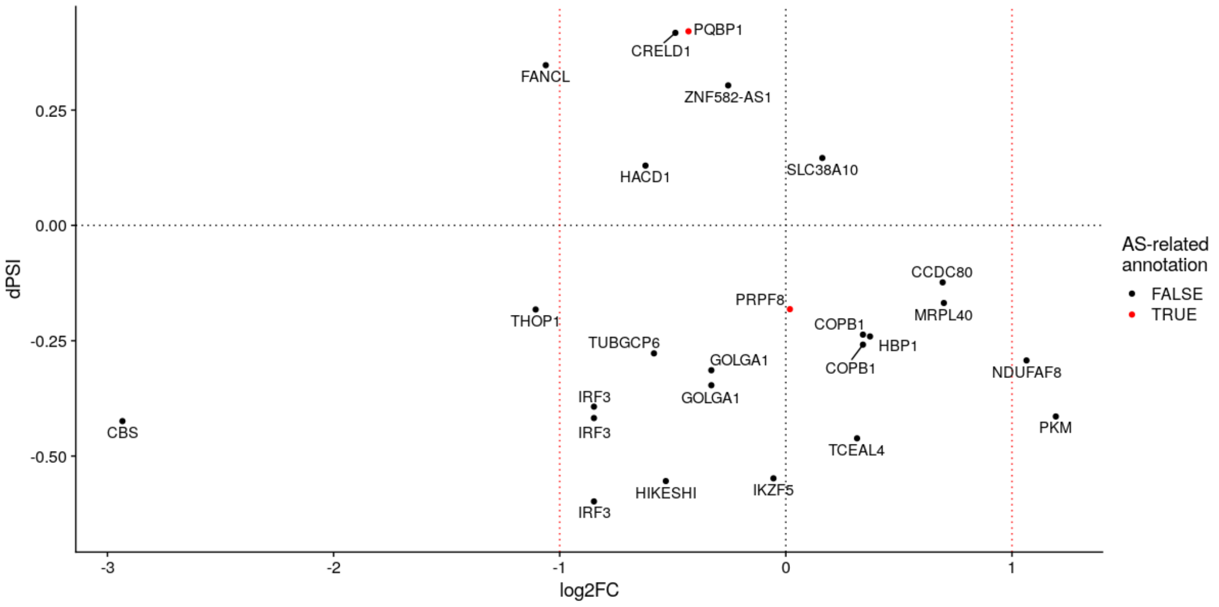

Supplementary Figure 8. Significant alternative 5' site events in the cell type contrast. Significantly AS alternative 5' are found in 30 genes. Comparison of dPSI (dPSI < 0 indicates event inclusion favored in Control, bottom panel; dPSI > 0 indicates event inclusion favored in Patient, upper panel) vs log2(Fold Change) (FC < 0: gene downregulated in patient, left panel; FC > 0: gene upregulated in patient, right panel).

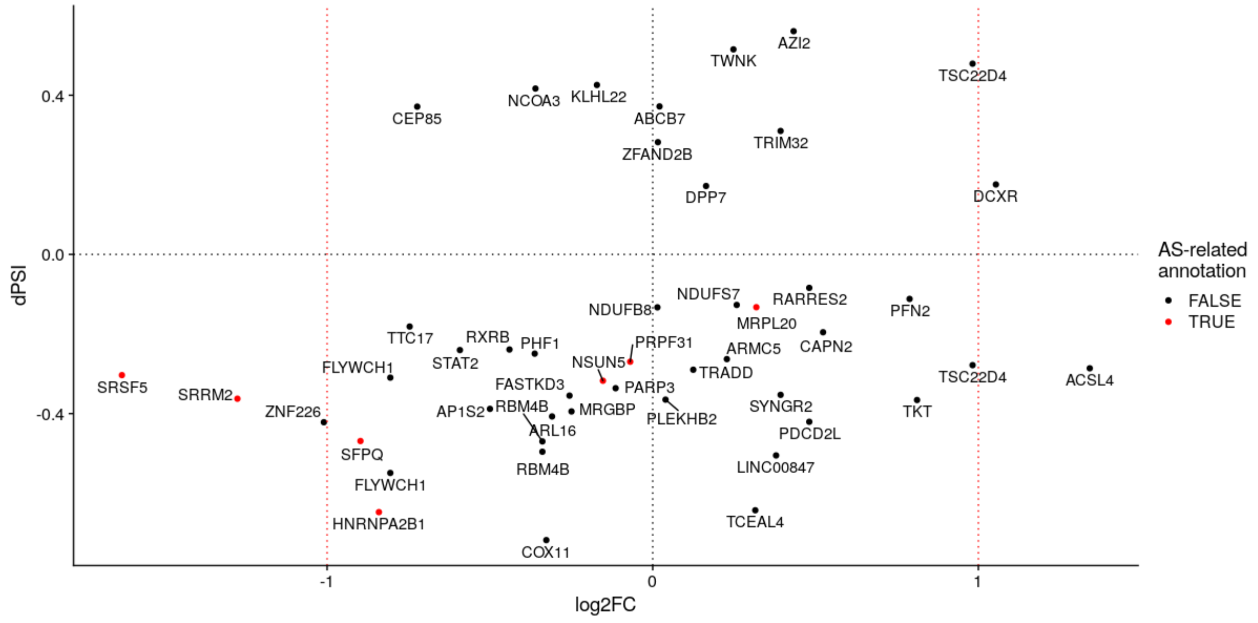

Supplementary Figure 9. Significant alternative 3' events in the cell type contrast. Significantly AS alternative 3' are found in 54 genes. Comparison of dPSI (dPSI < 0 indicates event inclusion favored in Control, bottom panel; dPSI > 0 indicates event inclusion favored in Patient, upper panel) vs log2(Fold Change) (FC < 0: gene downregulated in patient, left panel; FC > 0: gene upregulated in patient, right panel).

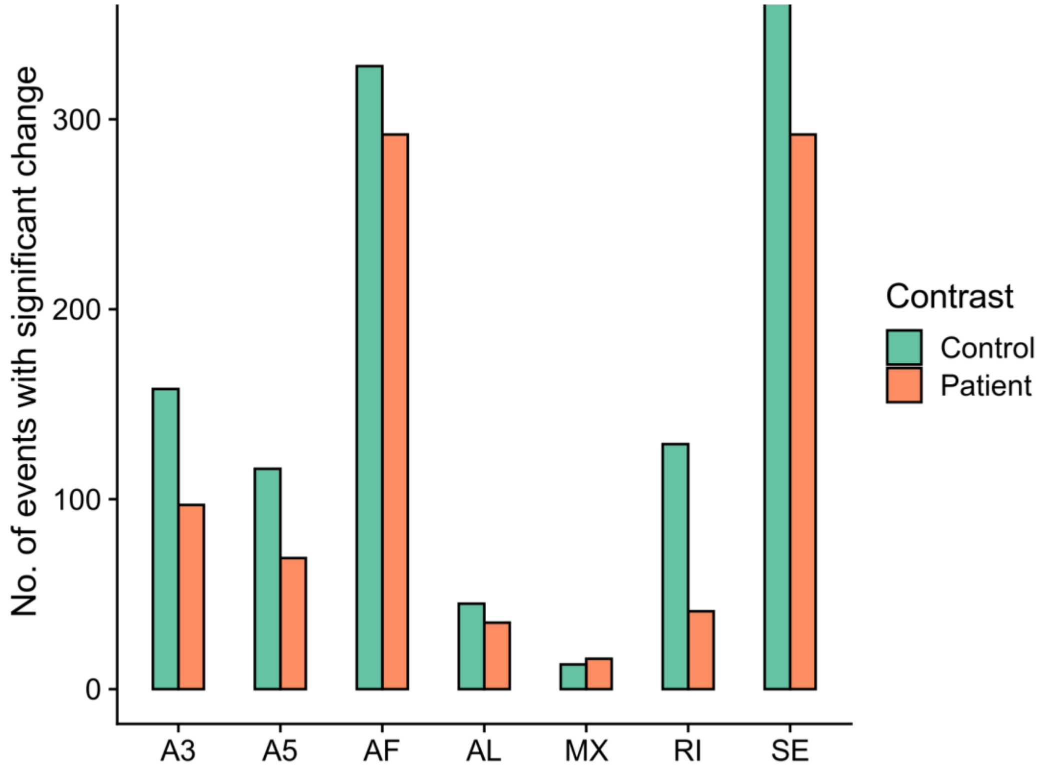

Supplementary Figure 10. Summary of significant alternatively spliced events detected in the within-condition contrasts.

The y-axis shows the number of significant splicing events ($p<0.05$) for each event category (x-axis), for the within-condition contrasts, i.e. control contrast (Control-fibroblasts vs Control-RPE) and patient contrast (Patient-Fibroblasts vs Patient-RPE). Event types: A3: Alternative 3' site; A5: Alternative 5' site; AF: Alternative Final exon; AL: Alternative Last exon; MX: Mutually-Exclusive Exons; RI: Retained Intron; SE: Skipped Exon.

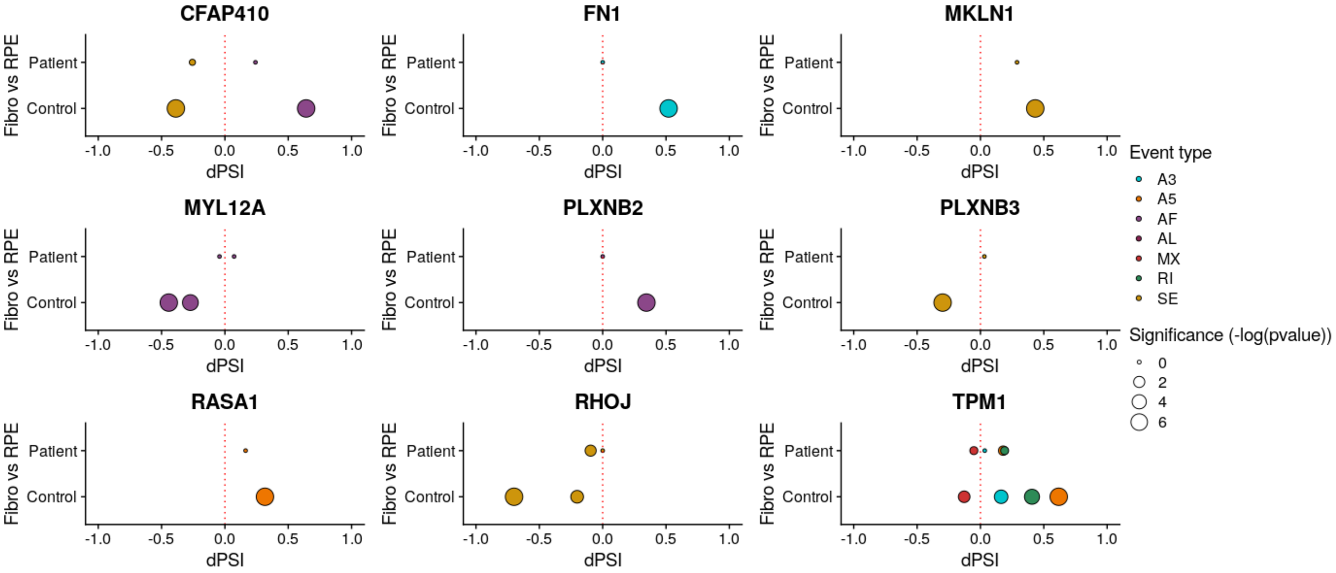

Supplementary Figure 11. Event state summary of unique differentially spliced genes in the control contrast (Control-RPE and Control-fibroblasts) annotated for *rRNA processing* (GO:0006364).

Point color represents the type of event. Point size represents significance (-log(p-value)) of the splicing change. y-axis indicates the contrast. x-axis represents dPSI (dPSI < 0: inclusion favored in fibroblasts; dPSI > 0: inclusion favored in RPE).

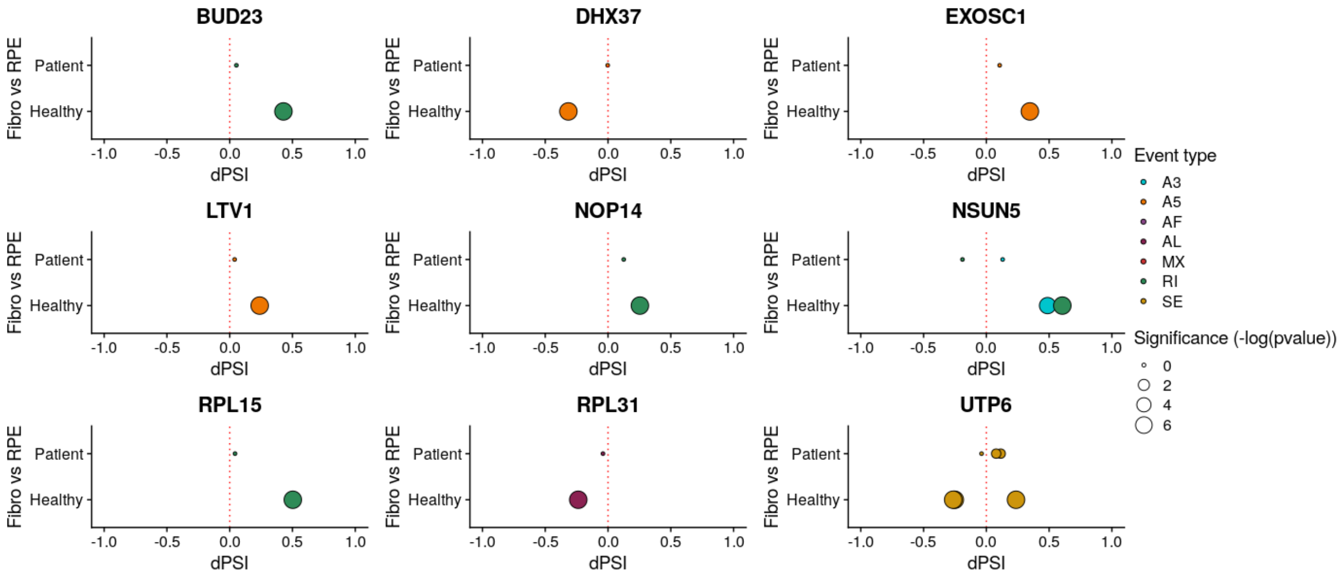

Supplementary Figure 12. Event state summary of unique differentially spliced genes in the control contrast (Control-RPE and Control-fibroblasts) annotated for *spliceosomal complex* (GO:0005681).

Point color represents the type of event. Point size represents significance (-log(p-value)) of the splicing change. y-axis indicates the contrast. x-axis represents dPSI (dPSI < 0: inclusion favored in fibroblasts; dPSI > 0: inclusion favored in RPE).

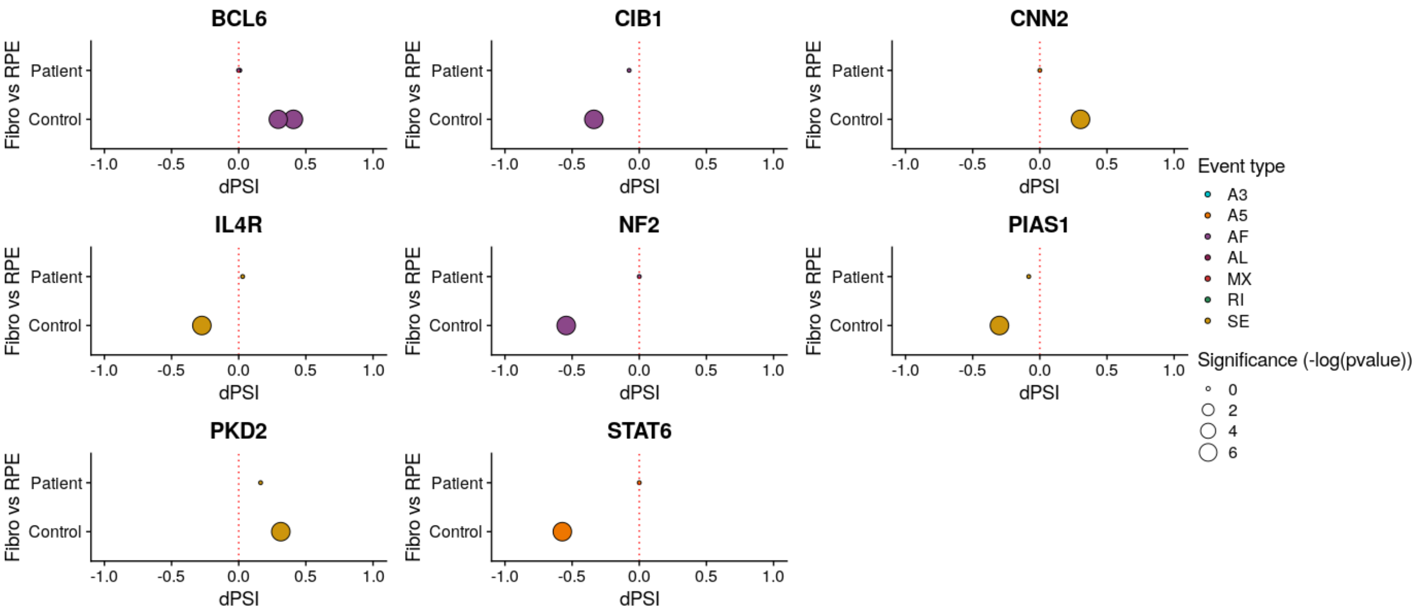

Supplementary Figure 13. Event state summary of unique differentially spliced genes in the control contrast (Control-RPE and Control-fibroblasts) annotated for *regulation of cell proliferation* (GO:0042127).

Point color represents the type of event. Point size represents significance (-log(p-value)) of the splicing change. y-axis indicates the contrast. x-axis represents dPSI (dPSI < 0: inclusion favored in fibroblasts; dPSI > 0: inclusion favored in RPE).

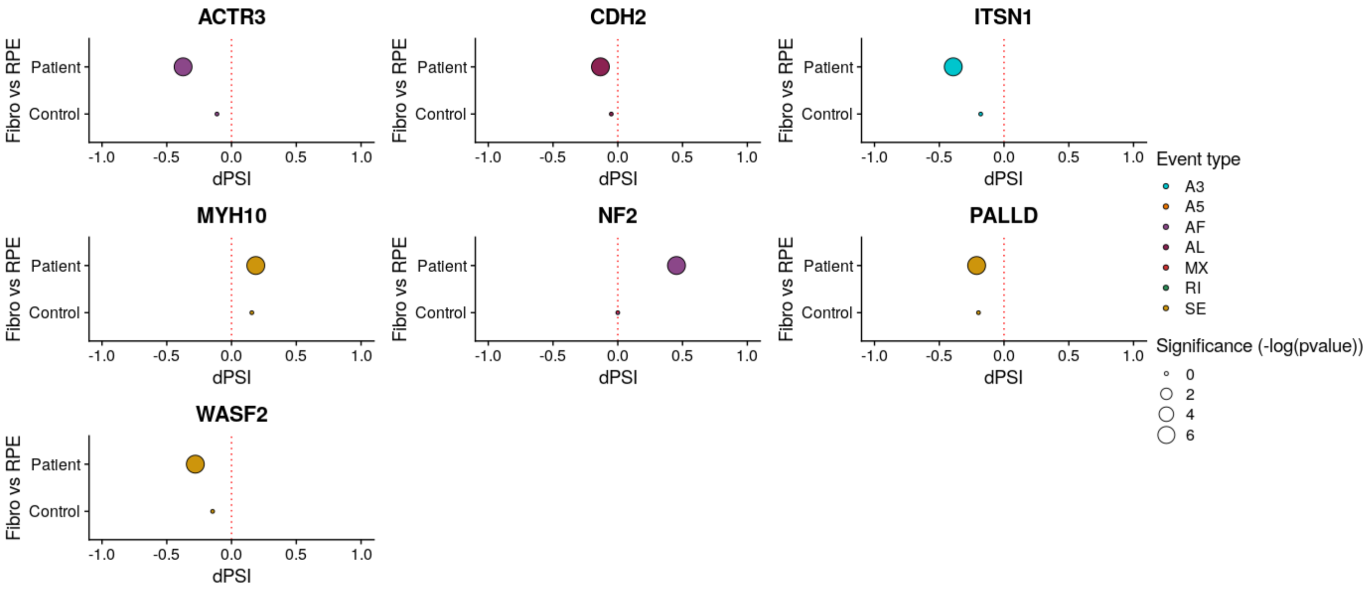

Supplementary Figure 14. Event state summary of unique differentially spliced genes in the patient contrast (Patient-RPE and Patient-fibroblasts) annotated for *regulation of cell proliferation* (GO:0042127).

Point color represents the type of event. Point size represents significance (-log(p-value)) of the splicing change. y-axis indicates the contrast. x-axis represents dPSI (dPSI < 0: inclusion favored in fibroblasts; dPSI > 0: inclusion favored in RPE).

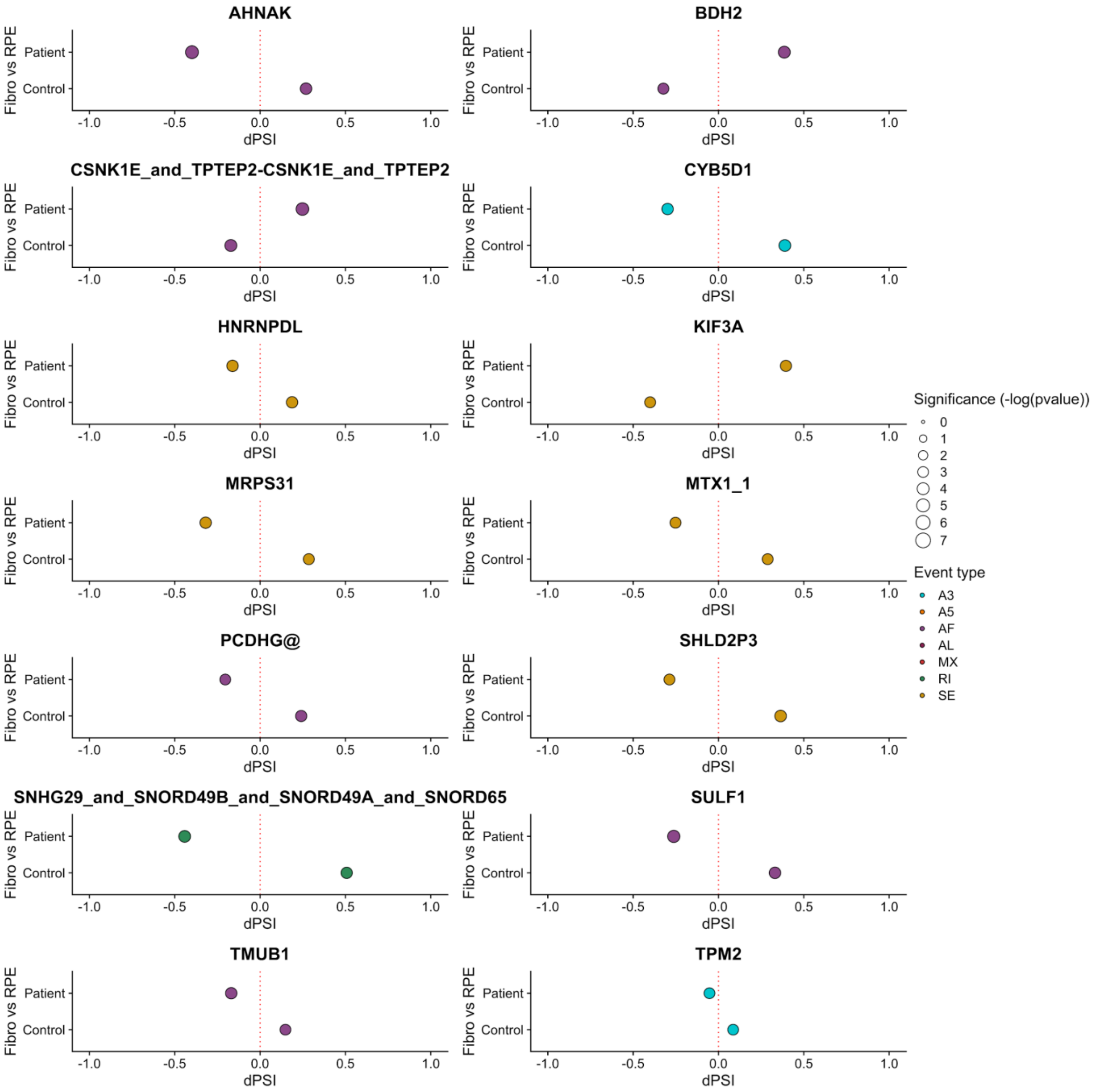

Supplementary Figure 15. Event state summary of differentially spliced genes common to the patient and control contrasts.

PCDHG@ corresponds to an event that is overlapping several genes from the PCDHG (protocadherin gamma) gene cluster. Point color represents the type of event, and point size represents the strength of the significance (-log(p-value)) of the splicing change between fibroblasts and RPE, for patient and control. We have represented both the patient and control cell type contrasts for comparison of the splicing state of the genes in each condition. dPSI < 0: inclusion favored in fibroblasts; dPSI > 0: inclusion favored in RPE.

Supplementary Tables

		Patient-Fibroblasts	Control-RPE
Control-Fibroblasts	All	2020	12394
	Unique	-	4903
Patient-RPE	All	9329	5391
	Unique	1838	4644

Supplementary Table 1. Differential Expression (DE) analysis results for all contrasts.

The number of significantly DE genes (p-value < 0.05) between each pair of conditions compared is shown. Unique DE genes after removing the different sources of interference (i.e. common genes with other contrasts, see Methods) are indicated between brackets.

Patient-RPE vs Control-RPE: unique events	Patient-Fibroblasts vs Control-Fibroblasts: unique events	Common events
329	198	32

Supplementary Table 2. Alternative Splicing (AS) analysis results: summary for within-cell type contrasts.

The number of common and unique significantly alternatively spliced events (p-value < 0.05) is shown. Common events between both contrasts were filtered to only retain unique events for downstream analysis.

Patient-RPE vs Patient-Fibroblasts: unique events	Control-RPE vs Control-Fibroblasts: unique events	Common events
471	784	371

Supplementary Table 3. Alternative Splicing (AS) analysis results: summary for within-condition contrasts.

The number of common and unique significantly alternatively spliced events (p-value < 0.05) is shown. Common events between both contrasts were filtered to only retain unique events for downstream analysis.

**iPSC line**	***PRPF8* gene**	**Clinical phenotype**	**Sex**	**Age at biopsy**
RP2-FiPS4F1	c.6974_6994del/WT<br>(Val2325_Glu2331del/WT)	RP	F	42
Ctrl1-FiPS4F1	WT	Healthy	M	40

Supplementary Table 4. Summary of patient and control information.
